# A whole genome Bayesian scan for adaptive genetic divergence in West African cattle

**DOI:** 10.1186/1471-2164-10-550

**Published:** 2009-11-21

**Authors:** Mathieu Gautier, Laurence Flori, Andrea Riebler, Florence Jaffrézic, Denis Laloé, Ivo Gut, Katayoun Moazami-Goudarzi, Jean-Louis Foulley

**Affiliations:** 1 INRA, UMR de Génétique Animale et Biologie Intégrative, 78350 Jouy-en-Josas, France; 2 University of Zurich, Institute of Social and Preventive Medicine, Zurich, Switzerland; 3 CEA, Centre National de Génotypage, 91057 Evry, France

## Abstract

**Background:**

The recent settlement of cattle in West Africa after several waves of migration from remote centres of domestication has imposed dramatic changes in their environmental conditions, in particular through exposure to new pathogens. West African cattle populations thus represent an appealing model to unravel the genome response to adaptation to tropical conditions. The purpose of this study was to identify footprints of adaptive selection at the whole genome level in a newly collected data set comprising 36,320 SNPs genotyped in 9 West African cattle populations.

**Results:**

After a detailed analysis of population structure, we performed a scan for SNP differentiation via a previously proposed Bayesian procedure including extensions to improve the detection of loci under selection. Based on these results we identified 53 genomic regions and 42 strong candidate genes. Their physiological functions were mainly related to immune response (MHC region which was found under strong balancing selection, CD79A, CXCR4, DLK1, RFX3, SEMA4A, TICAM1 and TRIM21), nervous system (NEUROD6, OLFM2, MAGI1, SEMA4A and HTR4) and skin and hair properties (EDNRB, TRSP1 and KRTAP8-1).

**Conclusion:**

The main possible underlying selective pressures may be related to climatic conditions but also to the host response to pathogens such as *Trypanosoma(sp)*. Overall, these results might open the way towards the identification of important variants involved in adaptation to tropical conditions and in particular to resistance to tropical infectious diseases.

## Background

Cattle are still playing a major role in Africa for food supply, to generate income and draught power or for ceremonial purposes. Archaeological, historical and anthropological evidence combined with recent genetic data [1] have provided insights into the complex origins of present day West-African cattle diversity. Indeed, although their wild ancestor *Bos primigenius *was not native to sub-Saharan Africa, West African cattle populations are representative of both shorthorn (*Bos taurus brachyceros*) and longhorn (*Bos taurus longifrons*) humpless taurines, humped zebus (*Bos indicus*) and zebu/taurine hybrid cattle. This early suggested that West African cattle has originated from several successive and recent colonization events [2,3]. Briefly, shorthorn taurines were introduced from the Middle-East and possibly North Africa around 4,000 years BP [3,4] while longhorn taurine probably arrived at an earlier period (5,000 years BP) following different migration routes [3]. Although, zebu cattle first penetrated through the Horn of Africa in the late 2^nd ^millennium BC, the major wave of indicine introgression really started with the Arab settlements along the East Coast of Africa from the end of the 7^th ^century AD. Zebu cattle spread even more recently over West Africa with movements of pastoralist people such as the Fulani [1].

**Figure 1:**
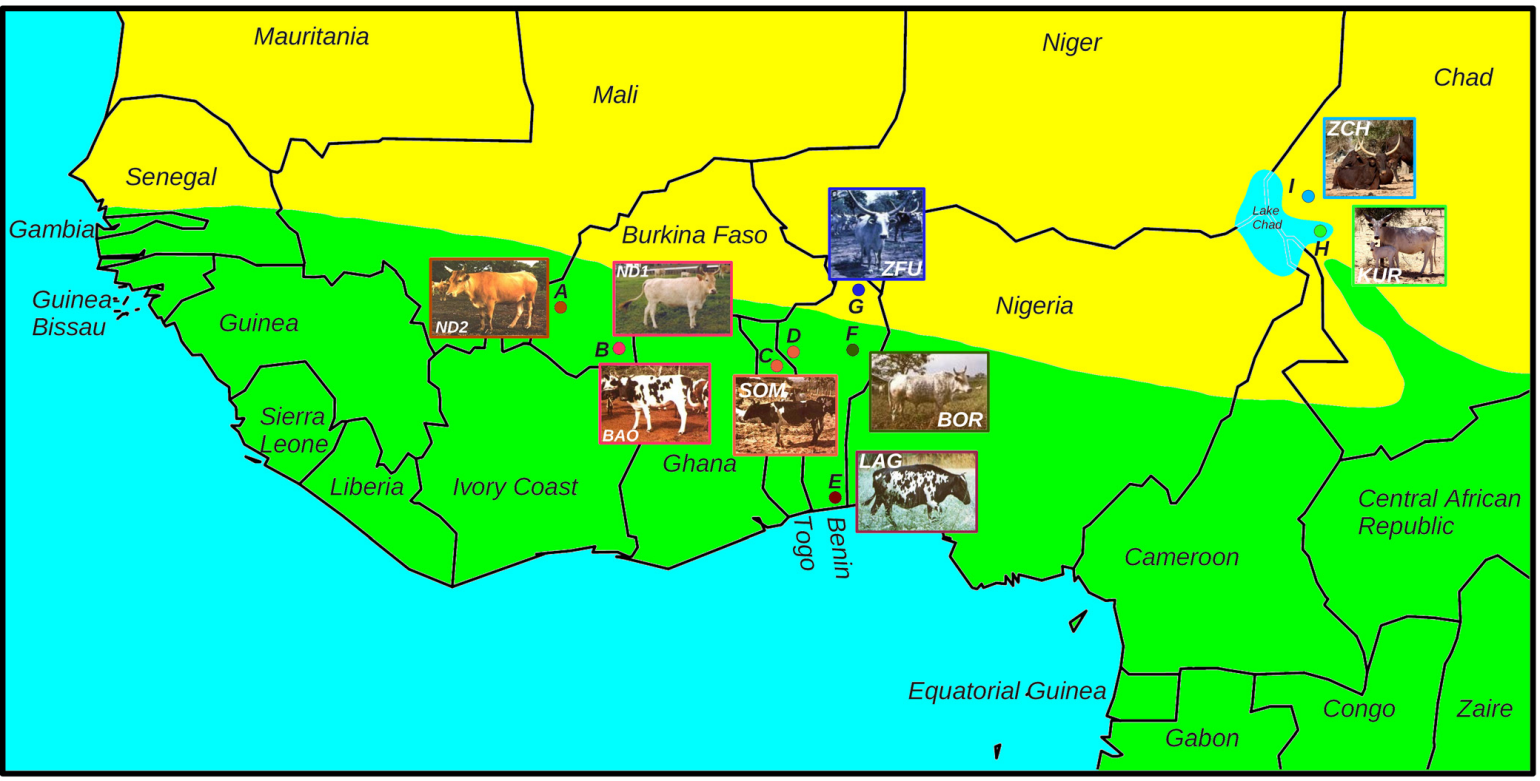
**Origin of the West African population samples**. A) N'Dama ND2 samples (n = 17) originated from the Samandeni ranch in Burkina Faso [64]; B) Baoulé (BAO) samples (n = 29) and N'Dama ND1 (n = 14) originated from the Gaoua Ranch in Burkina-Faso [64]; C) and D) Somba (SOM) samples (n = 44) originated from Boukombe (Benin) and Nadoba (Togo) regions [65]; E) Lagune (LAG) samples (n = 44) originated from the Porto Novo region in Benin [65]; F) Borgou (BOR) samples (n = 45) originated from the Parakou district in Benin [65]; G) Sudanese Fulani (ZFU) samples (n = 43) originated from the Malanville region in Benin [65]; H) Kuri (KUR) samples (n = 47) were collected in Lake Chad islands [64] and I) Choah zebu (ZCH) samples originated from the Bol district in Chad [64]. The tsetse infested region is colored in green on the map.

As a consequence of their remote origin, West African cattle populations have been subjected in recent times to new environmental pressures imposing strong adaptive constraints [5]. Indeed, tropical climate conditions might have affected several traits such as reproduction, grazing behavior, feed/water intake and utilization, milk production and growth. For instance, some West African shorthorn cattle which are exposed to very harsh conditions have been subjected to a marked reduction in size [3]. In addition, cattle were exposed to new pathogens in particular parasites. A well described example of newly acquired adaptation to parasitic disease is the ability, known as trypanotolerance, of taurine cattle to survive, reproduce and remain productive within the tsetse infested sub-tropical zone characterized by a high prevalence of trypanosomiasis (Figure 1) [6]. This might have in turn limited the introgression in these areas of zebus which are trypanosusceptible (Figure 1).

West African cattle populations thus represent an appealing model to unravel the genome response to adaptation to tropical conditions. The purpose of this study was to perform a whole genome scan for footprints of adaptive selection based on a newly collected genotyping data set containing 36,320 SNPs genotyped on 9 West African cattle populations from different bovine sub-species and agro-ecological areas (Figure 1). In particular, we sampled populations on both side of the tsetse infested zone. Based on this large data set, we first carried out a detailed analysis of the genetic structure of these populations. We next performed a scan for differentiation among SNPs under a full Bayesian framework [7,8] for which we proposed additional extensions to improve the detection of loci under selection. We then annotated regions containing SNPs subjected to selection, adopting a systems biology approach to highlight the main underlying physiological functions.

## Results and Discussion

### SNP polymorphism

In total, 437 individuals belonging to 12 different cattle populations, nine of which originating from West Africa (Figure 1), were genotyped for the BovineSNP50 chip assay containing 54,001 SNPs mainly derived from sequences available in European cattle breeds [9]. Among the autosomal SNPs that passed Quality Control analyses (see Methods), we retained the 36,320 SNPs polymorphic (Minor Allele Frequency or MAF above 0.01) in at least one West-African taurine and one West African zebu populations. As expected and shown in Figure S1A (additional file 1), this SNP selection procedure, leading mainly to the elimination of SNPs from European origin, resulted in a relative increase in calculated heterozygosity for all but outgroup taurine populations (Aubrac or AUB and Oulmès Zaer or OUL). In addition, as revealed by the distribution of the MAF for the different populations (Figure S1B in additional file 1), the remaining differences in heterozygosity among populations were mainly explained by the proportion of SNPs with rare variants (MAF < 0.05). In particular, marker polymorphism remained clearly lower in West African taurine populations (ND1, ND2, LAG, SOM and BAO), the proportion of SNPs with a MAF < 0.05 being the highest (36%) in LAG which also displayed the lowest average heterozygosity (0.118). These observations are in agreement with previous studies based on microsatellite markers on the same populations [10] and might essentially be explained by the recent demographic history of West African taurine cattle characterized by a marked isolation associated with a strong effective population size decrease (*e.g. *[11]). Although West African zebus (ZFU and ZCH) and hybrids (BOR and KUR) displayed more genetic diversity than West African taurines in our study (Figure S1 in additional file 1), ZFU, ZCH and ZMA levels of polymorphism were clearly lower than, and BOR and KUR ones similar to the taurine outgroup (AUB and OUL) ones (Figure S1A in additional file 1). Nevertheless, studies based on microsatellite data (*e.g. *[10]) revealed that heterozygosities of ZCH, ZFU, BOR and KUR were higher than European breeds' ones (AUB) which were themselves similar to ZMA. The lack of zebu specific sequences in the SNP discovery process might explain these apparent discrepancies.

### Analysis of Population Structure

**Figure 2:**
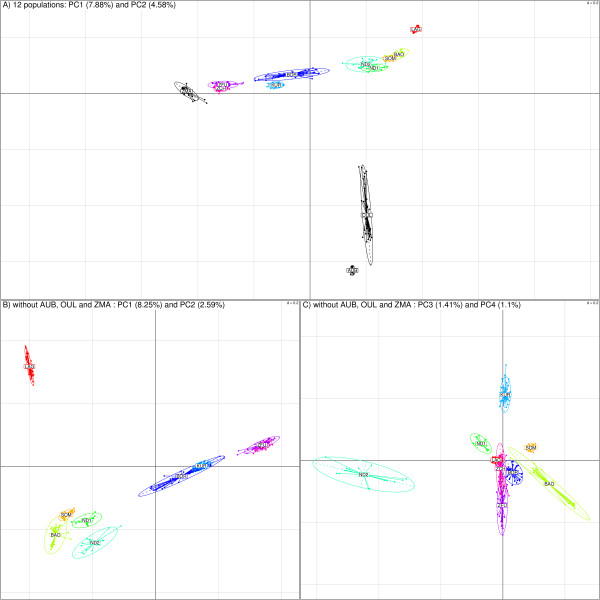
**PCA results**. Plots of the individuals according to their coordinates on the first two principal components on the eigenanalysis with (A) and without (B) the three outgroup populations (AUB, OUL and ZMA). Plots on the third and fourth components for this latter analysis are shown on Figure 2C. Ellipses characterize the dispersion of each breed around its center of gravity (assuming the cloud is a random sample distributed according to a bivariate gaussian distribution, the probability for an individual to be within the ellipse is 0.9).

The neighbor-joining tree based on Allele Sharing Distances (ASD) [12] allowed us to unambiguously separate individuals according to their population of origin (Figure S2 in additional file 1). West African individuals branched in their expected intermediary position relatively to the taurine (OUL and AUB) and zebu (ZMA) outgroups. Moreover, West African zebus (ZFU and ZCH) were closer to ZMA while West African taurines (LAG, ND1, ND2, BAO and SOM) were closer to OUL and AUB. KUR and BOR individuals branched between West African taurines and zebus. To go further in the characterization of the population relationships we carried out a principal component analysis (PCA) [13] based on all available SNP information [14]. As shown in Figure 2A and in agreement with previous published studies [1,4,15,16], the first component which accounted for 7.88% of variation separated West African populations according to a zebu/taurine gradient while the second (accounting for 4.58% of the total variance) could be associated to an Africa/Europe gradient, the North African OUL being closer to the European AUB. A similar PCA performed after removing the three outgroup populations (ZMA, OUL and AUB) allowed to distinguish West African populations according to the same zebu/taurine gradient on the first axis (which accounted for 8.25% of the total variance). The second and third components (which accounted respectively for 2.59% and 1.41% of the total variance) separated respectively LAG and ND2 from the other West African taurines (BAO, ND1 and SOM) (Figure 2B). The position of ND1 along the third axis suggested a certain level of admixture with shorthorn populations, most probably of BAO origin, according to their sampling area (Figure 1), although pairwise *F*_*ST *_divergence was smaller when compared to SOM (Table S1 in additional file 2). Finally, the fourth component (which accounted for 1.10% of the total variance) separated both West-African hybrids (BOR and KUR) and zebus (ZFU and ZCH) (Figure 2C). Overall, these results demonstrated a clear partition of the West African populations considered. *F*_*ST *_between pairs of populations (Table S1 in additional file 2) were all found significantly non null (P << 0.00001) and ranged between 0.013 (for ZFU compared to ZCH) and 0.28 (for LAG compared to ZFU).

As reported in Table S1 (additional file 2), within population *F*_*IS *_were almost null (< 0.006) for ZFU and BOR or very close to zero (from -0.0121 to 0.0198) for ND1, BAO, KUR and ZCH. The slightly positive *F*_*IS *_(0.0421) observed in SOM might result from the sampling area being apart the border between Togo and Benin. The positive *F*_*IS *_(0.0414) of similar magnitude observed in LAG might rather originate from a higher level of inbreeding as suggested by the extent of Linkage Disequilibrium (LD) within this population (see below). Conversely, for ND2, the negative *F*_*IS *_(-0.108) might be explained by recent outcrossing events. Indeed ND2 individuals derived from founders recently collected in different and distant Ivorian villages (see Methods). This hypothesis is also in agreement with their higher dispersion compared to other West African taurines in the PCA (Figure 2) and a higher level of overall extent of LD (see below). Nevertheless, the global *F*_*IS *_coefficient remained close to zero (0.0103) while the global *F*_*ST *_and *F*_*IT *_were respectively equal to 0.132 and 0.141. Such a level of differentiation among the West African populations was in agreement with the one computed based on microsatellite markers [10] suggesting a low impact of the SNP ascertainment procedure on these estimates.

### Bayesian scan for differentiation among SNPs

We further studied the differentiation among SNPs and populations based on a Bayesian hierarchical model derived from [7] and presented in more details in the Methods section. Among the factors influencing population differences in allele counts (differentiation), the model aims at distinguishing locus-specific from population-specific factors, such as migration or drift [17]. Because most SNPs originated prior to breed differentiation, the effect of different mutation rates across loci might be negligible and the SNP-specific effect in the model is mainly related to selection. Notice that the model assumed the *F*_*IS *_to be null within each population which appeared sound given the characteristics of our data (see Figure S3 in additional file 1 and Methods). We also ignored ascertainment biases originating both from the SNP discovery process and our selection of SNPs "informative" in West African populations. These two different sources are expected to favour polymorphic SNPs of ancient origin (co-segregating in European and African cattle breeds) and thus modifies to some extent the distribution of allele frequencies in the gene pool (*x *following our model notations) [18]. Recently, efficient algorithms were proposed to account for ascertainment biases [19], providing it can be modelled (which is difficult in our case at least for the first identified source). Nevertheless, for the purpose of this study we were mainly concerned with possible biases introduced in the locus-specific effect estimation which might not be of importance [18]. At least for our second source of ascertainment bias, simulated data allowed to confirm this statement (Figure S4 in additional file 1) since estimation of *x *was found robust to departure from the prior distribution assumed. Similarly, although more variable, locus-specific effect estimates were highly correlated with their corresponding simulated values (r > 0.8).

**Figure 3:**
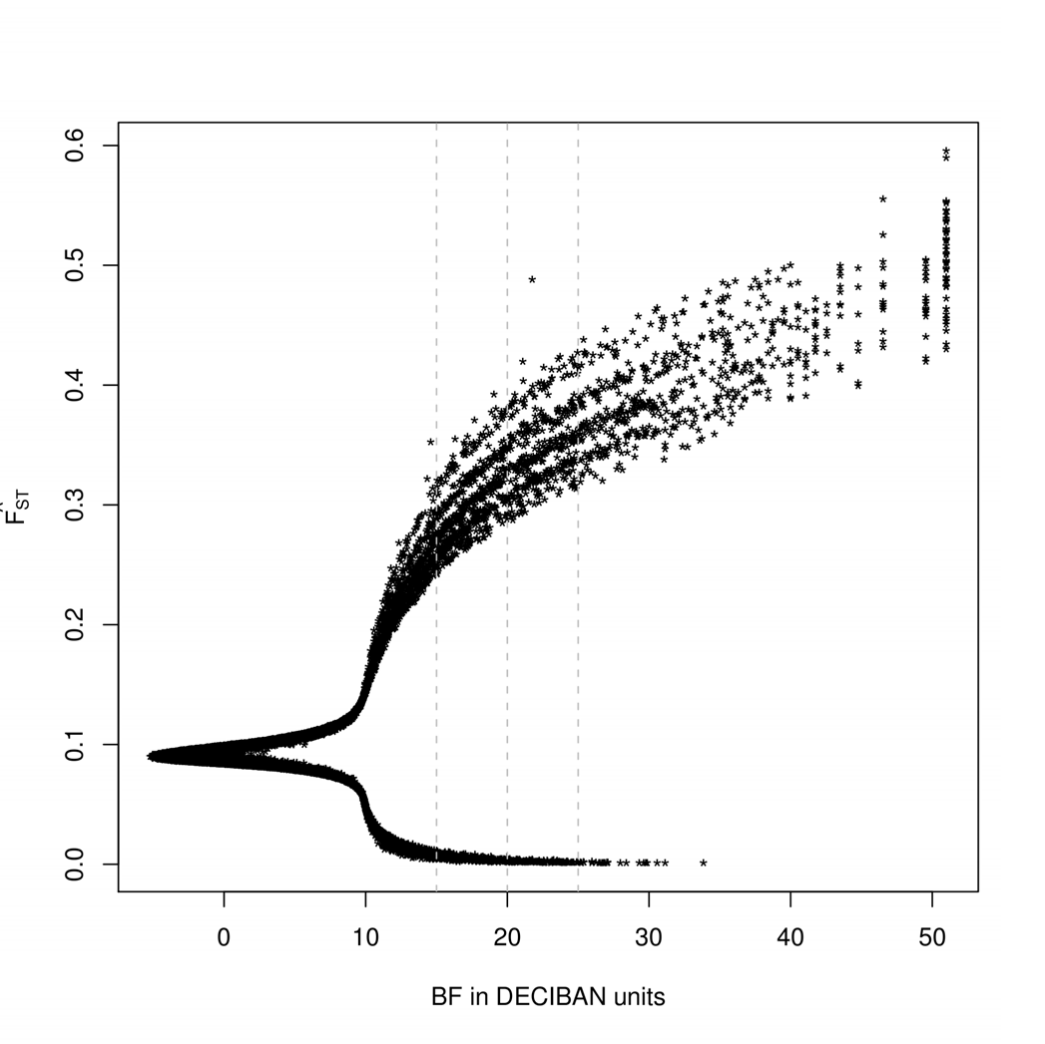
**Plot of the estimate of the locus *F*_*ST *_against Bayes Factor**. The three dashed lines represent the 15, 20 and 25 BF threshold.

To identify loci subjected to selection we thus focused on the posterior estimates of the SNP effect and evaluated the significance of its departure to the null value (expected under the neutral hypothesis). In Figure 3, we plotted the posterior SNP specific *F*_*ST *_estimate against its Bayes Factor (*BF*) expressed in deciban units (dB). *BF *compare models with and without the locus effect in terms of posterior to prior odds ratios, thus providing a basis for a decision rule to identify SNPs under selection (see Methods). The mean (standard deviation) of the posterior distribution for the proportion *P *of SNPs under selection (computed as the average over loci of the indicator variable *δ*_*i *_associated to the SNP effect in the hierarchical model) was equal to 0.186 (2.7 × 10^-3^). Analyses of four simulated data sets (with respectively 0.1%, 1%, 10% and 18.6% of the SNPs under selection) revealed that such estimates were extremely robust to the prior distribution of *P *(Figure S5 in additional file 1). The high proportion of SNPs under selection confirmed that selection had a non negligible role in the differentiation of these populations.

To provide insights into the decision rule criterion (in terms of *BF *threshold) to declare SNPs as subjected to selection, we subsequently investigated the power and the robustness of the model using simulated data (see Methods). For each of the four previously mentioned simulated data sets, we estimated False Discovery Rates (FDR) and False Negative Rate (FNR) (Table 1). Application of the Jeffreys' rule (see Methods) proved efficient since the FDR, although slightly increasing with the simulated *P*, was always less than 0.5% when considering a threshold of 20 on BF (*i.e. *decisive evidence). However, for such stringent a threshold, the FNR was very high across the four simulated data sets (>80%), a detailed inspection (Table S2 in additional file 2) showing that most non discovered SNPs were those with the smallest simulated effects (*α*_*i *_following our notations). More precisely, for SNPs under positive selection, the FNR was less than 30% (<10%) when *α*_*i *_>2 (*α*_*i *_>4) while above 98% when *α*_*i *_<1. In addition, as expected from previous studies [7,8,20] and from our overall relatively low level of differentiation, the model was less powerful to detect SNPs under balancing selection. The observed FNR remained high even for very large (in absolute value) *α*_*i *_(FNR>60% when *α*_*i *_<-4). Decreasing the threshold to a less stringent value such as 15 ("very strong evidence" according to Jeffreys' rule) reduced substantially the FNR (FNR<31% when *α*_*i*_<-4 and FNR<51% when *α*_*i *_<-2) and increased only slightly the FDR. To illustrate how these values of *α*_*i *_can be related to a coefficient of selection *s *we finally performed simulations under a Wright-Fisher island model with selection for various *s *and types of selection [20]. For SNPs under positive selection, *α*_*i *_>2 was achieved for loci with *s *>0.05 (under a dominant model) while for SNPs under balancing selection *α*_*i *_remained low even for large *s *(*α*_*i *_= -1.40 for s = 0.5) (Table 2). This confirmed that given our overall level of differentiation, the approach is expected to be powerful for positively selected SNPs with moderate to high effect but might lack some power even for those under high balancing selection.

**Table 1:** FDR (FNR) for different thresholds on BF.

BF threshold (in dB units)	P = 0.1%	P = 1%	P = 10%	P = 18.5%
0	1.40 (70.0)	2.98 (56)	14.0 (37.9)	28.5 (27.6)
5	0.477 (78.0)	1.07 (66.2)	4.75 (50.1)	8.84 (44.4)
10	0.166 (84.0)	0.364 (75.4)	1.71 (61.1)	3.23 (55.7)
**15**	**0.0501 (88.0)**	**0.158 (82.6)**	**0.653 (71.3)**	**1.23 (66.1)**
**20**	**0.0180 (88.0)**	**0.0788 (86.2)**	**0.231 (79.9)**	**0.418 (76.2)**
**25**	**0.0120 (88.0)**	**0.0384 (87.8)**	**0.100 (84.3)**	**0.172 (82.1)**
30	0.0120 (88.0)	0.0242 (88.4)	0.0578 (86.9)	0.0934 (85.4)
35	0.0120 (90.0)	0.0242 (89.4)	0.0400 (88.7)	0.0369 (87.3)
40	0.00 (100)	0.0182 (89.6)	0.0333 (89.5)	0.0197 (88.1)
45	0.00 (100)	0.0141 (90.0)	0.0333 (89.8)	0.0172 (88.5)
50	0.00 (100)	0.0141 (90.0)	0.0311 (89.9)	0.0172 (88.7)

Results are presented for four simulated data sets with a varying proportion *P *of loci under selection.

**Table 2:** Relationship between selection coefficient (*s*) and locus effect *α*_*i*_

Simulated *s*	Number of loci simulated	Average FST (sd)	FST range (median)	*α*_*i*_
0	4663	0.128 (0.06)	0.007-0.479 (0.121)	0
0.0025	395	0.173 (0.085)	0.019-0.479 (0.166)	0.355
0.005	371	0.189 (0.105)	0.012-0.646 (0.171)	0.465
0.0075	362	0.21 (0.108)	0.025-0.56 (0.196)	0.594
0.01	343	0.223 (0.113)	0.024-0.585 (0.211)	0.671
0.02	287	0.327 (0.152)	0.019-0.729 (0.348)	1.199
0.03	270	0.439 (0.183)	0.041-0.748 (0.489)	1.673
0.04	258	0.509 (0.194)	0.068-0.841 (0.572)	1.954
0.05	276	0.527 (0.199)	0.026-0.838 (0.588)	2.029
0.075	252	0.57 (0.242)	0.018-0.887 (0.677)	2.199
0.1	247	0.649 (0.227)	0.068-0.925 (0.728)	2.534
0.5	192	0.71 (0.239)	0.04-0.972 (0.801)	2.816
0.0025	486	0.12 (0.052)	0.006-0.339 (0.113)	-0.071
0.005	498	0.11 (0.049)	0.017-0.305 (0.104)	-0.167
0.0075	500	0.104 (0.047)	0.014-0.269 (0.096)	-0.24
0.01	500	0.091 (0.044)	0.002-0.267 (0.086)	-0.383
0.02	500	0.07 (0.039)	0.005-0.286 (0.061)	-0.669
0.03	500	0.056 (0.036)	0.006-0.238 (0.048)	-0.907
0.04	500	0.051 (0.037)	0.002-0.177 (0.043)	-1.005
0.05	500	0.05 (0.034)	0.004-0.203 (0.042)	-1.02
0.075	500	0.041 (0.033)	0.001-0.19 (0.031)	-1.226
0.1	500	0.04 (0.034)	0.001-0.22 (0.029)	-1.263
0.5	500	0.035 (0.034)	0-0.187 (0.027)	-1.403

### A Genome map of regions under adaptive differentiation

**Figure 4:**
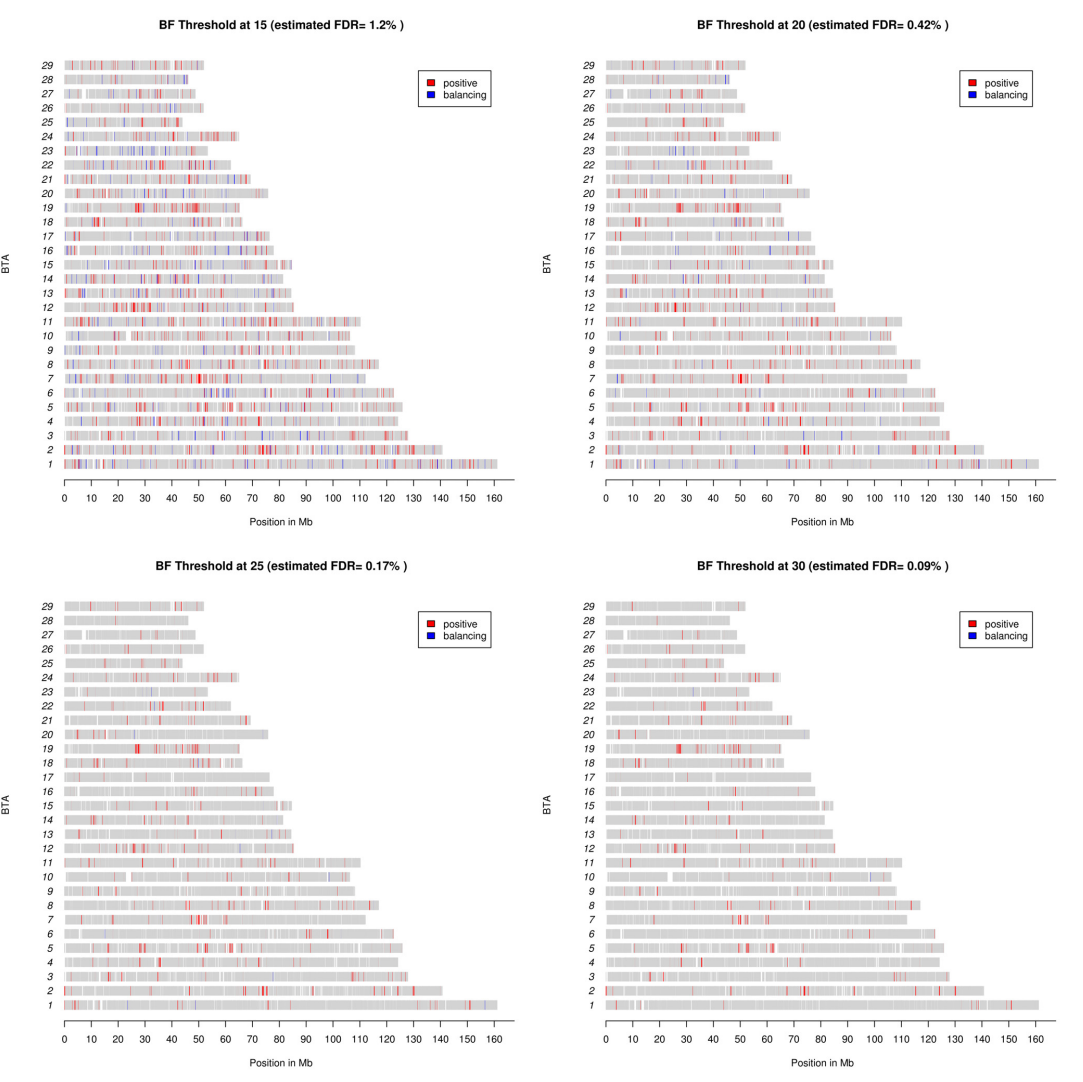
**Genome position of the locus declared under selection**. For each BF thresholds, the color indicates whether the SNP is neutral (grey), under positive selection (red) or balancing selection (blue). The type of selection (balancing or positive) is deduced from the SNP *F*_*ST *_value (< 0.1 or > 0.1).

Whole genome maps of adaptively differentiated loci in West African populations are given in Figure 4 for four different thresholds on *BF *value. In total, 2,054 (5.7%), 1,119 (3.1%), 619 (1.7%) and 375 (1.0%) of the 36,320 SNPs displayed a *BF *above 15, 20, 25 and 30 respectively (Table S3 in additional file 2). As expected (see above) most of the SNPs displaying high evidence of selection were highly differentiated suggesting they were subjected to positive selection (Figures 3 and 4). More precisely, among the SNPs with a BF value above 15, 537 were under balancing selection with a *F*_*ST *_< 0.011 while 1517 were clearly under positive selection with a *F*_*ST *_> 0.28. At a more stringent threshold on BF of 30, 3 SNPs remained under balancing selection (*F*_*ST *_< 0.0013) and 372 under positive selection (*F*_*ST *_> 0.33). Interestingly, the whole genome distribution of SNPs declared under selection appeared less uniform as the threshold on *BF *increased (Figure 4). This might be explained by the effect of selection which tends to increase LD between markers [21]. Yet, we did not include in the model any spatial structure, which may take into account the LD among SNPs, as recently proposed ().().(.)[22]. Within the different West African populations and at the distance of 70 kb corresponding to our average marker spacing (Figure S6 in additional file 1 and Methods), r^2 ^was close to the asymptotic value (<0.1 in all populations except ND2) (Figure S7 in additional file 1) which is related to the current effective population size [23]. As a result, the level of association between most SNP pairs was expected to be of similar magnitude to the one between unlinked markers as illustrated by the correlations of the estimated *F*_*ST *_and *BF *between pairs of SNPs which quickly dropped towards 0 as they were more distantly related (Table S4 in additional file 2).

**Figure 5:**
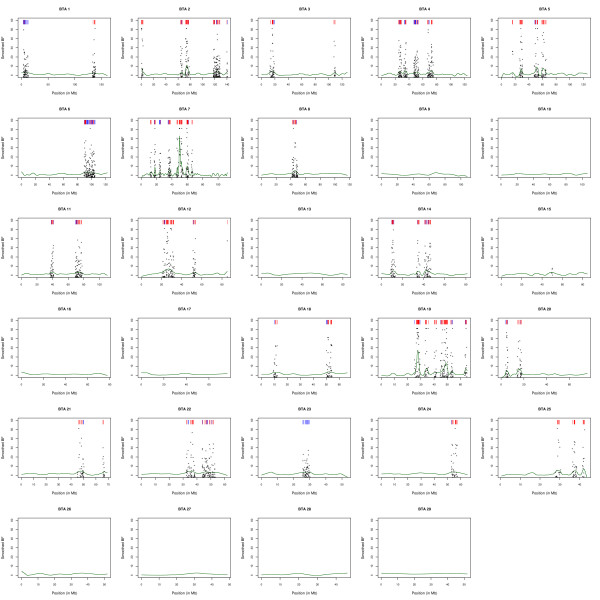
**Whole genome map of regions under selection at the 5% local FDR (q-value) threshold**. For each of the 29 bovine autosomes, the smoothed BF is plotted against the chromosomal position (green line). For significant positions, non smoothed SNP BF are indicated by a black star. At the corresponding positions, a red (blue) bar is represented on the top of each graph if the SNP was under positive (balancing) selection *i.e. *displayed a (non smoothed) BF > 15 and a *F*_*ST *_> 0.1 (*F*_*ST *_< 0.1).

As an attempt to identify those genomic regions with unexpectedly high proportions of selected SNPs, we smoothed individual SNP *BF *values over each chromosome (see Methods). This empirical, non parametric approach can efficiently combine information from several neighboring SNPs and allows the identification of significantly differentiated regions. This strategy might provide in turn a first empirical attempt to correct for false positives as expected when a hierarchical structure exists among populations under study *i.e. *when some populations share a recent ancestry or there are barriers to gene flow between some of them [24]. As shown in Figure 5 and Table 3, 53 significant regions were identified at the 5% local FDR (q-value) threshold. In agreement with strong hitchhiking effects, these regions together contained 49.9% of the SNPs with a BF larger than 30 and 24.9% of the SNPs with a BF larger than 15. In addition, most of these SNPs were subjected to positive selection (*F*_*ST *_> 0.1) although a notable exception is represented by the region on the middle of BTA23 which was exclusively composed of SNPs under balancing selection. Interestingly, this region contains BoLA, the bovine Major Histocompatibility Complex (MHC) for which balancing selection has already been extensively reported in other species [25,26] and also in other cattle populations [15]. To a lesser extent, a few other regions, such as the ones localized on the centromeric part of BTA01, the telomeric part of BTA06 and the middle of BTA22 (Figure 5) contained a high proportion of SNPs under balancing selection. This might be related to the maintenance of several haplotypes containing variants under positive selection within different populations. Alternatively, the fixation of the same variant in some populations could also lead to such a trend because of the low level of LD across populations [11].

**Table 3:** Regions under selection identified at the 5% local FDR (q-value) thresholds.

Interval	BTA	Position (size) in Mb	Peak Position in Mb	BF value of the SNP at the peak position (q-value of the smoothed signal)	Gene at or close to the peak
1	1	3.654-12.74 (9.087)	3.923	50 (0.0467)	KRTAP8-1 (3.888-3.888)
2	1	133.1-138.6 (5.465)	138.1	51 (0.0345)	BFSP2 (138.1-138.2)
3	2	0.035-2.687 (2.652)	0.035	51 (0.0001)	NA
4	2	64.04-67.28 (3.235)	64.25	31 (0.0258)	CXCR4 (64.22-64.22)
5	2	71.48-78.32 (6.84)	73.84	51 (0.0001)	SNRPG (73.75-73.75)
6	2	117.7-129.0 (11.35)	124.1	45 (0.0007)	DIS3L2 (123.844-124.15)
7	2	140.0-140.6 (0.662)	140.0	25 (0.0473)	PADI4 (139.9-139.9)
8	3	12.39-19.72 (7.325)	15.96	51 (0.0007)	SEMA4A (15.94-15.96)
9	3	107.9-110.1 (2.219)	108.3	37 (0.0246)	RNF220 (108.2-108.3)
10	4	24.74-29.70 (4.953)	25.47	38 (0.0002)	NA
11	4	32.31-37.17 (4.860)	35.71	36 (0.0009)	NA
12	4	46.84-54.10 (7.259)	51.86	26 (0.0017)	EPDR1 (51.77-51.81)
13	4	66.29-75.27 (8.977)	67.67	50 (0.0065)	NEUROD6 (67.70-67.70)
14	5	15.625-16.745 (1.121)	16.15	26 (0.0342)	NA
15	5	26.81-31.05 (4.245)	28.37	51 (0.0001)	PDE1B (28.34-28.39)
16	5	47.89-54.39 (6.505)	49.93	51 (0.0004)	DYRK2 (49.89-49.90)
17	5	58.82-65.26 (6.44)	61.43	47 (0.0001)	RBMS2 (61.40-61.46)
18	6	89.36-105.4 (16.05)	98.15	51 (0.0011)	ANTXR2 (98.00-98.24)
19	7	11.59-13.35 (1.753)	12.98	25 (0.0176)	OLFM2 (12.90-12.99)
20	7	17.02-18.86 (1.846)	17.90	51 (0.0001)	TICAM1 (17.90-17.90)
21	7	23.53-25.51 (1.987)	25.24	24 (0.0036)	CTXN3 (25.23-25.24)
22	7	34.96-38.66 (3.703)	36.52	28 (0.0001)	NA
23	7	46.17-54.47 (8.296)	49.91	51 (0.0001)	MATR3 (49.89-49.93)
24	7	58.19-61.94 (3.745)	59.43	51 (0.0001)	HTR4 (59.31-59.50)
25	7	65.23-67.12 (1.897)	66.43	28 (0.0005)	RPL35A (66.46-66.46)
26	8	41.46-49.56 (8.100)	43.54	51 (0.0011)	RFX3 (43.24-43.56)
27	11	36.82-41.39 (4.565)	41.08	33 (0.0346)	NA
28	11	69.22-77.83 (8.614)	72.17	38 (0.0126)	ALK (71.89-72.63)
29	12	20.68-32.60 (11.93)	23.08	51 (0.0009)	NA
30	12	51.02-54.06 (3.033)	53.56	40 (0.0265)	EDNRB (53.54-53.57)
31	12	85.24-85.28 (0.034)	85.24	37 (0.0498)	CDC16 (85.23-85.25)
32	14	9.072-13.53 (4.461)	11.38	50 (0.0017)	NA
33	14	33.32-36.43 (3.108)	34.44	38 (0.0022)	NA
34	14	40.36-46.85 (6.481)	46.08	35 (0.007)	TRPS1 (46.06-46.34)
35	15	48.98-50.50 (1.522)	50.01	8 (0.0451)	TRIM21 (50.07-50.08)
36	18	9.24-12.36 (3.119)	12.17	32 (0.0457)	CLDN9 (12.17-12.17)
37	18	49.99-54.41 (4.412)	51.08	41 (0.0128)	CD79A (51.08-51.08)
38	19	25.14-29.43 (4.283)	26.87	51 (0.0001)	MINK1 (26.86-26.91)
39	19	32.87-35.72 (2.857)	33.74	51 (0.0008)	TEKT3 (33.71-33.74)
40	19	40.02-41.82 (1.798)	41.72	51 (0.0401)	CASC3 (41.70-41.72)
41	19	44.62-54.41 (9.792)	49.62	51 (0.0001)	FTSJ3 (49.61-49.62)
42	19	63.36-64.95 (1.594)	64.26	51 (0.0051)	CCDC46 (64.16-64.31)
43	20	4.040-6.339 (2.299)	4.745	47 (0.0008)	ERGIC1 (4.632-4.745)
44	20	13.97-18.06 (4.093)	16.50	37 (0.0016)	RNF180 (16.31-16.56)
45	21	45.09-50.35 (5.261)	46.29	51 (0.0174)	KIAA0391 (46.20-46.33)
46	21	65.61-66.89 (1.279)	65.74	30 (0.0324)	DLK1 (65.72-65.72)
47	22	32.32-38.56 (6.235)	36.54	47 (0.0137)	MAGI1 (36.14-36.79)
48	22	43.79-53.04 (9.249)	51.81	34 (0.0451)	CCDC71 (51.80-51.80)
49	23	25.59-30.12 (4.529)	29.06	27 (0.0235)	NA
50	24	52.78-57.25 (4.466)	55.71	51 (0.0255)	NA
51	25	27.73-30.38 (2.653)	28.70	51 (0.0102)	SRCAP (28.67-28.70)
52	25	36.33-38.47 (2.142)	37.28	37 (0.0013)	MYL2 (37.32-37.33)
53	25	41.40-42.52 (1.115)	42.52	50 (0.0278)	AMZ1 (42.51-42.53)

### Functional annotation of regions under selection

The 36,320 SNPs were representative of about 7,177 different genes corresponding to approximately one third of the total number expected in the genome. For each of these, Table S5 (additional file 2) summarizes the number of SNPs they contained and results from the differentiation analysis. Individual SNP information made it nevertheless difficult to propose a list of genes subjected to selection and this strategy might be more sensitive to possible false positives introduced by hierarchical structure among the populations under study [24]. Alternatively, a great proportion of such genes could include SNPs appearing as significantly differentiated as a result of hitchhiking with a favourable variant located nearby (see above). Hence, among the 191 (853) genes containing or close to at least one SNP with a BF > 30 (BF > 15), 103 (237) mapped within one of the 53 regions identified above. For functional and network analyses, we thus decided to mainly concentrate on the 42 genes which were located for 25 of them (represented in Table S5 in additional file 2), under the peak of each region, or for the remaining 17 less than 50 kb from the peak of each region (Table 3) (and not represented by any of the 36,320 SNPs surveyed). These 42 genes were viewed as the strongest candidates underlying the observed footprints of selection. Among these, 37 genes were eligible for network analysis, 29 being also eligible for functional analysis. The five remaining ones (CCDC46, CTXN3, KRTAP8-1, RNF180, TEKT3) were not eligible for any network or functional analyses.

**Figure 6:**
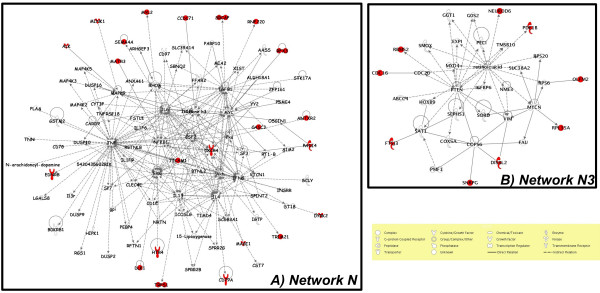
**Representation of the N (A) and N3 (B) gene networks**. Symbols corresponding to candidate genes are colored in red.

Four significant networks namely N1, N2, N3 and N4 (Table S6 in additional file 2) were identified. Three of them (N1, N2 and N4) were interconnected and further merged into a single network (N). Networks N and N3 are represented in Figure 6 and their functional annotations detailed in Table S7 (additional file 2). The main hubs of N corresponded to genes encoding cytokines (CSF2, IFNG, IL4, IL13, IL6, TGFB1 and TNF) and protein kinases (Akt and Erk). Network N contained 22 of our candidate genes (ALK, ANTXR2, CAC3, CCDC71, CD79A, CXCR4, DLK1, DYRK2, EDNRB, HTR4, MAGI1, MATR3, MINK1, MYL2, PADI4, RFX3, RNF220, SEMA4A, SRCAP, TICAM1, TRIM21 and TRPS1), some of them being involved in innate and adaptive immune response (Figure 6A, Table S7 in additional file 2). Network N3 contained molecules mainly involved in cancer, cellular function and maintenance, and neurological disease, and among them nine candidate genes (CDC16, DIS3L2, FTSJ3, NEUROD6, OLFM2, PDE1B, RBMS2, RPL3A and SNRPG). Six other candidate genes (AMZ1, BSFP2, CLDN9, EPDR1, ERGIC1 and KIAA0391) were eligible for network analysis but were included neither in N nor N3. Overall, the functional annotation suggested that the three main physiological functions targeted by selection in the breeds we studied were related to i) immune response, ii) nervous system and iii) skin and hair development which we discuss in the following.

### Immune response genes under selection

Several candidate genes (such as CD79A, CXCR4, DLK1, RFX3, SEMA4A, TICAM1 and TRIM21) belonging to N directly participate in antigen recognition, a key process underlying the development of immune response. For instance, TRIM21, a newly identified type of IgG Fc receptors [27] participates to both the host anti-viral response through the modulation of IRF3 functions [28] and mediation of autoimmune diseases. Footprints of selection were recently reported in primates within another member of the same tripartite motif (TRIM) family of proteins which has antiretroviral properties [29,30]. TICAM1 is a Toll like receptor (TLR) adapter and thus plays a role in innate immunity. It possesses, in particular, a high IFN type I inducing activity during viral infection [31]. DLK1, a member of the epidermal growth factor-like gene family, has been shown in mouse to be involved in B lymphocytes differentiation and function [32]. More specifically, other genes (such as CD79A, CXCR4, RFX3 and SEMA4A) are related to antigen presentation to T cells in the context of MHC. Although no MHC genes were present in the list analyzed, the corresponding region (#49 in Table 3) and a SNP (with *BF *= 17 and *F*_*ST *_= 0.005) within the bovine HLA-DMB ortholog (Table S4 in additional file 2) were found under strong balancing selection. Among the molecules participating to antigen presentation, the chemokine receptor CXCR4 is co-recruited with CCR5 in the immunological synapse, and it participates to modulation of T cells response [33]. These two molecules were shown to act as co-receptors for HIV entry in human cells [34]. A strong signature of balancing selection was reported in the 5' cis-regulatory region of CCR5 in human [35], this region displaying as well a selective sweep in chimpanzee [36]. We also recently observed a significant signal of selection on a microsatellite located less than 1 Mb from CXCR4 in West African taurine [10] and defining the boundary of a QTL underlying the trypanotolerance trait in cattle [37]. Similarly, RFX3 (43.235-43.563 Mb on BTA08) is located within another QTL identified in this latter study [37]. This member of the regulatory factor X (RFX) gene family participates with CIITA in the regulation of MHC genes [38-40]. CD79A via CD79*α*-CD79*β *heterodimer located on the surface of B cells is required for antigen presentation through MHC class II [41]. SEMA4A a semaphorin expressed on dendritic cells and B cells is involved in T cell priming and Th1 differentiation through its interaction with Tim2 expressed on T cells [42].

Although heat stress response may have imposed immunological parameter modifications [5], infectious and parasitical diseases are likely to have represented selective pressures acting on genes involved in such functions [43]. As previously mentioned, trypanosomiasis represents one of the well known examples of such selective pressure. Nevertheless, the importance of other diseases such as tick-borne diseases (anaplasmosis, babesiosis, cowdriosis) or anthrax should not be overlooked. For instance, anthrax is hyperendemic (especially in Chad, Togo and Ivory Coast) or endemic in West Africa http://www.vetmed.lsu.edu/whocc/mp_world.htm. Notice that the ANTRXR2, a receptor of the anthrax toxin was among our candidate genes (see network N, Figure 6).

### Neural genes under selection

In addition to the exposure to new pathogens, the recent settlement of cattle to Sub Saharan Africa might have imposed a dramatic change in their environmental conditions [5]. Hence, cattle (in particular taurine) were exposed to warmer temperature, to a thermal amplitude decrease, to a day length change, to differences in solar radiation and also to new feeding conditions. As a result, adaptations needed modifications in global appearance (such as morphology or coat color), in body temperature regulation, in circadian clock and also in reproductive behaviour and abilities. Such selective pressures might partly explain the physiological functions related to nervous system (neurogenesis and eyesight). Alternatively, neurotropism of several parasites such as *Trypanosoma(sp) *have been demonstrated at least in human. Some of the candidate genes related to nervous system development and function belonged to N3 (e.g. NEUROD6 and OLFM2) or N (e.g. MAGI1, SEMA4A, HTR4 and EDNRB). NEUROD6 is a member of the neurogenic differentiation transcription factor family [44] while OLFM2 is a secreted glycoprotein belonging to the noelins family which modulate the timing of neuronal differentiation during development [45]. Interestingly, MAGI1 is a scaffolding protein present in tight junction of epithelial cells [46], some transcripts of the corresponding gene being only expressed in brain. Moreover it is located within a QTL underlying the trypanotolerance trait [37]. Besides their role in immune response (see above), semaphorins represented by SEMA4A were initially characterized for their role in the guidance of axonal migration during neuronal development. Mutations in the conserved semaphorin domain of SEMA4A are associated with two retinal degenerative diseases, retinis pigmentosa and cone-rod dystrophy [47]. Of more particular interest is HTR4 which is a serotonin receptor participating to the serotonergic system. Recently, a mutation within HTR2A, a receptor of the same family was reported associated with the chronic fatigue syndrome corresponding to a dysregulation of hypothalamic-pituitary-adrenal (HPA) axis and serotonergic system [48]. A reduced responsiveness of the HPA axis after corticotropin-releasing hormone (CRH) challenge was observed in Boran cattle infected with *Trypanosoma congolense *[49]. Taken together, a possible involvement of HTR4 in trypanotolerance/susceptibility in cattle might be hypothesized though we cannot exclude an implication of this serotonin receptor in more general aspects of circadian rythmicity [50].

### Genes under selection involved in skin and hair properties

Although EDNRB plays an essential role in the development of enteric neurons, it is also involved in the development of epidermal melanocytes, both cell lineages being derived from the neural crest [51]. As an illustration, several EDNRB mutations are associated with an auditory pigmentary syndrome caused by the absence of melanocytes [52]. However, given the variety of cattle populations surveyed (Figure 1), the footprint of selection observed in the EDNRB region is more likely related to differences in coat color and more particularly to the spotting pattern (SOM, BAO and LAG) or the white color (KUR and ZFU). Indeed, EDNRB is also referred to as *piebald *or S locus in mouse [53] and a null mutation induce a white coat color in rat [51]. In the Hereford cattle breed, although not yet fully characterized, locus S seems responsible for white spotting pattern [53]. In addition to EDNRB involved in skin and hair pigmentation, other genes such as TRSP1 which belonged to N and KRTAP8-1 which was not eligible for network analysis play a role in hair development in human and mouse. Indeed, TRPS1 is a zinc finger transcription factor implicated in growth and trichosis, some of its variants being associated with trichorhinophalangeal syndromes [54,55]. It is down-regulated in patients affected by hypertrichosis and in a mouse model of hypertrichosis [56]. KRTAP8-1, a keratin associated protein, plays a role in the formation of hair shafts [57].

The skin color and thickness, the hair size and sleekness and the number of sweat glands directly influence thermo-resistance of cattle living in the tropics [5]. Compared to European taurine breeds, zebus which have a higher density of sweat glands and a smoother and shinier hair coat were reported to better regulate body temperature and more efficiently maintain cellular function during heat stress [58]. Similarly, slick-haired Holstein cattle are more able to regulate their body temperature than wild-type [59]. Some skin and hair properties might also confer higher resistance to tick infestation [60,61]. Interestingly, some genes involved in hair development also participate in cornification. Hence the observed footprint of selection within the cluster of keratin associated proteins (represented in the candidate genes list by KRTPA8-1, the closest to the peak) might thus also be related to horn morphology differences [62]. A striking example is the floating horns of KUR which could represent original adaptation facilitating swimming in their swamp living area [3]. Note that the identified region is also localized less than 5 Mb from the bovine polled locus [63]

## Conclusion

West African cattle provide a valuable resource to better understand the genomic response to selective pressure arising in tropical conditions. This large-scale whole genome Bayesian scan for adaptive differentiation in populations representative of the current breed diversity allowed us to identify candidate genes involved in several key physiological functions. These results might open the way towards the identification of variants underlying these footprints of selection, in particular those involved in the resistance to tropical infectious diseases in cattle but also in other mammals such as human populations subjected to similar pressures.

## Methods

### Animal Material

Figure 1 provides information on the origin of the nine different West African populations sampled in our study and representatives of the three main types of cattle found in this area. Longhorn taurines were represented by N'Dama individuals from two distinct origins (ND1 and ND2). ND1 individuals (n = 14) originated from the Gaoua herd constituted by acquisitions coming from different villages in the Pays Lobi (Burkina Faso). ND2 (n = 17) samples were collected in the Samandeni herd (Burkina Faso) which was constituted in the early eighties by acquisitions from the Marahoue ranch (Ivory Coast) and originated from northwestern villages from Ivory Coast where zebu had not been introduced [64]. Shorthorn taurines were represented by three different breeds: Baoulé (BAO) samples (n = 29) originated, as ND1, from the Gaoua herd [64], Somba (SOM) individuals (n = 44) were sampled in the breed birthplace across the border near Nadoba (Togo) and Boukoumbe (Benin), and Lagune (LAG) individuals (n = 44) were sampled in the Porto Novo district (Benin) [65]. West African zebus were represented by two populations: Sudanese Fulani or ZFU (n = 43) which was sampled in the Malanville region in Benin [65] and Choah zebus or ZCH (n = 59) which was sampled in the Bol district (Chad) [64]. This latter population was initially sub-divided into two breeds (M'Bororo and Choah Zebus), however, as previously shown [10] and confirmed in our analysis with a dense marker set, no clear differentiation appeared among these. Finally, two hybrid populations were considered: Borgou or BOR (n = 45) which is a stabilized crossbred between shorthorn taurines (LAG or BAO) and was sampled in its region of origin around Parakou in Benin [65] and Kuri or KUR (n = 47) which was sampled from different islands of Lake Chad around Bol [64]. This latter breed was sometimes referred to as a particular longhorn taurine [3], however, several molecular analyses showed a high level of Zebu admixture in it [4,10]. Moreover, unlike West African taurines, KUR is known to be trypano-susceptible. Three other breeds were considered as outgroups for the detailed genetic structure analysis: ZMA (n = 35) sampled in the Madagascar Island [66] and representing pure zebu [1], AUB (n = 20) sampled in the birthplace of the breed [67] and representing European taurine, and OUL (n = 40) sampled in the North of Morocco to represent North African taurine cattle.

### Genotyping data

Individuals were genotyped on the Illumina BovineSNP50 chip assay [9] at the Centre National de Génotypage (CNG) platform (Evry, France) using standard procedures http://www.illumina.com. Among the 54,001 SNPs included in the chip, only the 51,581 mapping to a bovine autosome on the latest bovine genome assembly Btau_4.0 [68] were retained for further analysis. To limit ascertainment bias favouring SNPs from European origin, we subsequently discarded 13,786 SNPs (~25%) which were not polymorphic (MAF > 0.01) in at least one West African taurine (ND1, ND2, LAG, SOM or BAO) and one West African zebu (ZCH or ZFU). Among the remaining ones, 1,422 SNPs which were genotyped on less than 85% of the individuals from at least one of the nine West African breeds, were also eliminated. An exact test for Hardy-Weinberg Equilibrium (HWE) [69] was subsequently carried out within each breed separately. Based on the obtained p-values, q-values [70] were estimated for each SNP using the R package qvalue http://cran.r-project.org/web/packages/qvalue/index.html. Fifty three SNPs with a q-value < 0.01 in at least one breed were then discarded from further analysis. In total, 36,320 SNPs were finally considered for the study leading to an average marker density of 1 SNP every 70 kb over the genome (Table S8 in additional file 2). Moreover, as shown in Figure S6 (additional file 1) and detailed in Table S8 (additional file 2), the genome coverage was homogeneous with a median distance between consecutive SNPs equal to 47.54 kb. Few large gaps between SNPs were present since the 95^th ^(99^th^) percentile of this distance was 189 kb (385 kb), the largest gap localized on BTA10 being 2 Mb long. Conversely, less than 0.5% of the distances between successive SNPs were shorter than 20 kb. Genotyping data are given in additional file 3.

### Analysis of Population Structure and characterization of the extent of LD

ASD were computed for each pair of individuals using all available SNP information by a simple counting algorithm: for a given pair of individuals *i *and *j*, ASD was defined as 1-*x*_*ij *_where *x*_*ij *_represents the proportion of alleles alike in state averaged over all genotyped SNPs. A neighbor-joining tree was computed based on the resulting distance matrix using the R package APE [71]. We subsequently performed a PCA based on all available SNP information using the SMARTPCA software package [14]. As suggested by Patterson et al. [14], we performed LD correction by replacing individual SNP values with the residuals from a multivariate regression without intercept on the two preceding SNPs on the map, providing they were less than 200 kb apart. Results were further visualized using functionalities available in the R package ade4 [72]. The global F-statistics *F*_*IT*_, *F*_*ST *_and *F*_*IS *_were estimated respectively in the form of *F*, *θ *and f [73] using the program GENEPOP 4.0 [74]. GENEPOP 4.0 was also used to estimate diversity for each locus and population both within individuals and among individuals within a population. The within breed *F*_*IS *_was derived from the average of these two quantities over all the SNPs. In order to evaluate the reliability of the *F*_*IS *_estimates we computed the mean and standard deviation over 10,000 samples of 5,000 randomly chosen SNPs. *F*_*ST *_statistics between populations were estimated using both SMARTPCA [14] and GENEPOP [74] which are based on two different models of population divergence.

In order to characterize the extent of LD, we computed the *r*^2 ^measure [23] between each marker pair within each breed separetely using Haploview 4.1 [75].

### Bayesian Model to analyze Differentiation among SNPs

Individual genotyping data were modelled according to the reparameterized extension, recently proposed by Riebler et al. [8], of the initial Bayesian hierarchical model developed by Beaumont and Balding [7]. Briefly and in the bi-allelic SNP case, let *a*_*ij *_be the observed reference allele (defined arbitrarily) count in population *j *= 1, ..., *J *at locus *i *= 1, ..., *I*. The conditional distribution given the true (unobserved) allele frequency *p*_*ij *_in that population at that locus is assumed to be binomial with parameters *n*_*ij *_(twice the number of genotyped individuals in population *j *at locus *i*) and *p*_*ij*_: *a*_*ij *_| *p*_*ij*_~*Bin*(*n*_*ij*_, *p*_*ij*_) (1). Note that 1) implicitly assumes populations are in HWE or their respective inbreeding coefficients (*F*_*IS*_) are null. Non null *F*_*IS *_could be taken into account in the model by considering instead that the three possible genotypes are drawn from a multinomial distribution with parameters corresponding to the number of individuals genotyped and genotype probabilities: (1-*F*_*IS*_^*j*^)*p*_*ij*_^2 ^+ *F*_*IS*_^*j*^*p*_*ij*_; 2(1-*F*_*IS*_^*j*^)*p*_*ij*_(1-*p*_*ij*_) and (1-*F*_*IS*_^*j*^)(1-*p*_*ij*_)^2 ^+ *F*_*IS*_^*j*^(1-*p*_*ij*_) [18]. Nevertheless, for co-dominant markers such as SNP and given the range of *F*_*IS *_values in our study (see Results), the binomial distribution seems to be reasonable (Figure S3 in additional file 1). The second step assumes that the true allele frequencies *p*_*ij *_are themselves sampled from a Beta distribution: *p*_*ij *_| *λ*_*ij*_, *x*_*i*_~*Beta*(*λ*_*ij*_*x*_*i*_, *λ*_*ij*_(1-*x*_*i*_)) (2). This distribution relies on an infinite Wright island model involving mutation, drift and migration at its equilibrium state [76,77]. Under this model, *x*_*i *_might be interpreted as the frequency of the chosen reference allele at locus *i *in the gene pool from which each allele frequency *p*_*ij *_is sampled. The scaling parameter *λ*_*ij *_reflects the gene flow from the gene pool to population *j*. Under Wright's model, this parameter is specific to each population *j *but remains homogeneous over loci so that any departure from that property may indicate that locus *i *is no longer neutral. Note that under the Beta distribution, the first two moments can be simply expressed as *E(p*_*ij*_) = *x*_*i*_and *Var(p*_*ij*_) = *x*_*i*_(1-*x*_*i*_)(1+*λ*_*ij*_)^-1^. Actually (1+*λ*_*ij*_)^-1 ^can be identified as a *F*_*ST*_^*ij *^coefficient [17]. The next level of the model consists of specifying the distributions of *λ*_*ij *_(or *F*_*ST*_^*ij*^) and of *x*_*i*_. These frequencies are nuisance parameters and uncertainty about them will be taken into account by assigning to them non informative prior such as a *Beta(1,1) *distribution (*e.g. *[78]). Following Beaumont and Balding [7], the *λ*_*ij*_'s are modelled via a linear model on the logistic transformation of the *F*_*ST*_^*ij*^. Since F_*ST*_^*ij*^/(1-F_*ST*_^*ij*^) = 1/*λ*_*ij*_, we can write this model in terms of *η*_*ij *_= *log(F*_*ST*_^*ij*^/(1-*F*_*ST*_^*ij*^)) = -log(*λ*_*ij*_) as: *η*_*ij *_= *α*_*i*_+ *β*_*j*_+ *γ*_*ij *_where *α*_*i *_is a locus effect, *β*_*j *_a population effect and *γ*_*ij *_an error term corresponding to a departure of the logit of *F*_*ST*_^*ij *^from the additive decomposition. For theoretical and computational reasons, it is more efficient to consider the previous decomposition under a hierarchical Bayesian structure than under its basic form, and implement it as follows: *η*_*ij *_| *α*_*i*_, *β*_*j*_, *σ*_*γ*_^2^~_*iid*_*N*(*α*_*i *_+ *β*_*j*_, *σ*_*γ*_^2^) with *β*_*j *_| *μ*_*β*_, *σ*_*β*_^2^~_*iid*_*N*(*μ*_*β*_, *σ*_*β*_^2^) and *α*_*i *_| *σ*_*α*_^2^~_*iid*_*N*(0, *σ*_*α*_^2^). Introduction of additional levels by defining priors on the variance components *σ*_*α*_^2^, *σ*_*β*_^2 ^and *σ*_*γ*_^2 ^is straightforward but adds marginal gain in the estimates and requires high supplementary computational costs (Gautier and Foulley, unpublished data). We thus gave for these components the known values proposed initially by Beaumont and Balding (2004): *σ*_*α*_^2 ^= 1, *σ*_*β*_^2 ^= 3.24 and *σ*_*γ*_^2 ^= 0.25. The prior mean *μ *for the *β*_*j *_was also taken as -2.0. Recently, Riebler *et al. *[8] introduced in the above logistic model an auxiliary indicator variable *δ*_*i *_attached to each locus specifying whether it can be regarded as selected (*δ*_*i *_= 1) or neutral (*δ*_*i *_= 0). They demonstrated the efficiency of this approach for improving the power of the statistical procedure, particularly for the detection of loci under balancing selection. Under this reparameterized model, the previous parameters *α*_*i *_are written as: *α*_*i *_= *δ*_*i*_*α*_i _* with *α*_i _* | *σ*_α_*^2^~_*iid*_*N*(0, *σ*_α_*^2^). The model further assumes a Bernoulli distribution for the indicator *δ*_*i *_variable with parameter *P*: *δ*_*i *_| P ~*Bin(1, P)*. P is itself assumed to be *Beta *distributed to take into account uncertainty on this crucial parameter. Here we took *P ~Beta(0.2,1.8) *[8], thus assuming that a priori 10% of the loci are on average under selection, but within a very large range of possible values as the 95% credibility interval goes from almost 0 to 65%. Note that the value *σ*_α_*^2 ^can be derived from *σ*_*α*_^2 ^since by construction *σ*_*α*_^2 ^= *E(P) σ*_α_*^2 ^(hence *σ*_α_*^2 ^= 10).

The posterior distributions of the different parameters of interest were estimated via MCMC procedures as previously described [8] from 10,000 post burn-in samples (with a burn-in period of 3,000 iterations) and a thinning interval of k = 25. Convergence was checked using standard criterion. Parameter estimates for the *F*_*ST*_^*ij *^were taken as the median of the posterior distribution.

### Decision rule to identify non neutral loci

In order to identify outlier loci, we decided to base the decision rule on a Bayes Factor *BF*_*i *_defined as the ratio of the posterior to the prior odds that locus *i *is selected:

As *δ*_*i *_is an indicator variable, the prior and posterior probabilities that *δ*_*i *_= 1 are easy to compute since they reduce to expectations as shown below:

where *π(P) *is the density of the distribution of *P *defined above. To make interpretation of the *BF*_*i *_easier, we expressed them in deciban units (dB) *ie dB*_*i *_= 10*log*_10_(*BF*_*i*_) so that 10 dB corresponds to an odds ratio of 10, 20 dB to an odds ratio of 100, 30 dB to an odds ratio of 1,000 and so forth. We then applied the Jeffreys' rule [79] which quantifies the strength of evidence (here to consider a SNP as being under selection) based on *BF *using the following scale: "strong evidence" when *10*<*dB*_*i*_<*15*, "very strong evidence" when *15*<*dB*_*i*_<*20 *and "decisive evidence" when *dB*_*i*_>*20*. Notice that the BF captures the change in the odds in favor of *δ*_*i *_= 1 as we move from the prior to the posterior. In that way, it makes this criterion independent of the prior distribution of *δ*_*i *_and thus guarantees some robustness.

### Simulated data

Following Foll and Gaggiotti [20], four data sets each comprising 50,000 (unlinked) SNPs with respectively 9300 (P = 0.186), 5000 (*P = 0.1*), 500 (*P = 0.01*) and 50 (*P = 0.001*) under selection (*α*_*i *_≠ *0*) were simulated under the inference model. To mimic characteristics of our samples, we considered 9 populations with parameters *β*_*j *_equal to the ones estimated on the real data (mean of the corresponding posterior distribution). For each pair of simulated locus/population, we subsequently simulated the *η*_*ij *_parameter by adding to the corresponding *β*_*j*_, a value of *γ*_*ij *_sampled from a Gaussian distribution (*γ*_*ij*_*~N(0,0.25)*) and a value for *α*_*i *_equal to 0 if the locus was neutral or sampled from a Gaussian distribution (*α*_*i*_*~N(0,10)*) if the locus was under selection. The frequency *x*_*i*_in the ancestral population (or migrant gene pool) was sampled from a Beta distribution with parameters equal to 0.7. This is equivalent to assume the ancestral population was at equilibrium with *4Nμ *= *0.7 *where *N *is the effective population size and *μ *the mutation rate [80]. Based on these simulated values for *x*_*i*_and *η*_*ij *_we subsequently derived the *p*_*ij *_parameter of the binomial distribution from which the simulated observed *a*_*ij *_was sampled (taking *n*_*ij *_= 80 for all *i *and *j*). Only SNPs displaying MAF>0.01 in at least one simulated West-African taurine and one West-African zebu (identified according to the simulated *β*_*j*_) were conserved which led to the exclusion of few SNPs (displaying *α*_*i *_too high in general). To a first approximation, this also allowed the mimicking of the ascertainment bias introduced by the SNP selection criterion. The posterior distributions of the different parameters of interest were then estimated as described above from 1,000 post burn-in samples (with a burn-in period of 3,000 iterations) and a thinning interval of k = 25. *BF*_*i *_for each simulated loci were then computed (see above) and used to compute the (true) FDR and FNR for a given threshold. To investigate relationships between the coefficient of selection and the magnitude of the locus effect *α*_*i*_we performed simulations under a simplified version (without mutation) of the Wright-Fisher island model with selection proposed by Beaumont and Balding [7]. Briefly, 10 random mating populations with a constant size of 1,000 individuals and deriving from the same ancestral population were reproduced for 500 generations assuming a global *F*_*ST *_of 0.15. In total, 5,000 neutral SNPs and 500 SNPs for each investigated coefficient and mode of selection were simulated. Initial allele frequencies were drawn from a Beta distribution with both parameters equal to 0.7. At the end of the process, SNPs displaying a MAF <0.01 in at least 2 populations were discarded. Locus specific *F*_*ST *_were then computed following Weir and Cockerham [73]. Following Foll and Gaggiotti [20], for each type of selection considered, the corresponding average *F*_*ST *_was compared to the one obtained under neutrality (*α*_*i *_= *0*) to estimate the average SNP effect.

### Annotation of the SNPs

Because the annotation of the bovine genome is still sparse, the gene content information was derived from the TransMap cross-species alignments available in the UCSC Genome Browser http://genome.ucsc.edu/. For closer evolutionary distances, the alignments are created using syntenically filtered BLASTZ alignment chains, resulting in a prediction of the orthologous genes in cow. In total, 46,598 different RefSeq identifiers were anchored to the Btau_4.0 bovine genome assembly http://genome.ucsc.edu/. Considering that most consecutive SNPs on the map were separated by more than 20 kb and the relatively high level of LD at shorter distance (see Results and [11]) a SNP was considered as representative of a gene if it was localized within the boundary positions of the gene that extended by 15 kb upstream and downstream. According to this criterion, 15,360 out of the 36,320 SNPs were representative of 17,190 different TransMap RefSeq identifiers. Subsequent annotation and analyses were carried out with the Ingenuity Pathway Analysis (IPA) software v7.0 (Ingenuity Systems Inc., USA, http://www.ingenuity.com/). Among the 17,190 different TransMap RefSeq identifiers, 17,151 identifiers (99.7%) were represented in the Ingenuity Pathway Knowledge Base (IPKB) and corresponded to 7,177 different genes further considered as the reference set. Finally, 15,336 SNPs were located within a gene, leading to an average of 2.21 SNPs per annotated gene. Notice that 421 of these SNPs were representative of more than one gene.

### Functional and Network Analyses

Functional and Networks analyses were carried out using an approach similar to the one described in [81]. Based on the information contained in the IPKB, IPA allows performing both functional and network analyses. The functional analysis aims at identifying the most significant biological functions represented in the candidate gene list from the reference set. Note that among the genes under selection, only those associated with at least one functional annotation in IPKB were eligible for functional analysis. The most significant functions are then obtained by comparing functions associated with eligible genes against functions associated with all the genes in the reference set using the right-tailed Fisher's exact test. The network analysis aims at searching for interactions (known from the literature) between candidate genes and all other IPKB molecules (genes, gene products or small molecules) and result in the definitions of networks which contains at most 35 molecules (including candidate genes). For each network, a score *S *is computed based on a right-tailed Fisher exact test for the overrepresentation of candidate genes (*S *= -*log(p-value)*). In our study, networks were considered as significant when S>3 and their associated top functions and diseases were further reported.

### Identification of regions under selection

In order to identify regions under selection (with an unexpectedly high proportions of SNPs subjected to selection), we followed the locally adaptive procedure which allows to account for variations in distance between the different tested positions [81,82]. Individual SNP *BF *values were first smoothed over each chromosome with a local variable bandwidth kernel estimator [83]. We further performed 250,000 permutations to estimate local p-values which were corrected for multiple testing by computing q-values (see above).

## List of Abbreviations

*Throughout the text, gene symbols are given according to HUGO standard nomenclature*.

**Breed Codes**: AUB: Aubrac; BAO: Baoulé; BOR: Borgou; KUR: Kuri; LAG: Lagune; ND1: N'Dama (first population); ND2: N'Dama (second population); OUL: Oulmès Zaer; SOM: Somba; ZCH: Choah zebu; ZFU: White Fulani Zebu; ZMA; Zebu from Madagascar.

**Other abbreviations**: ASD: Allele Sharing Distance; BF: Bayes Factor; BTA: Bos TAurus chromosome; dB: deciban unit; HWE: Hardy-Weinberg Equilibrium; IPA; Ingenuity Pathway Analysis; IPKB: Ingenuity Pathway Knowledge database; FDR: False Discovery Rate; FNR: False Negative Rate; LD: Linkage Disequilibrium; MAF: Minor Allele Frequency; MHC: Major Histocompatibility Complex; PCA: Principal Component Analysis; SNP: Single Nucleotide Polymorphism.

## Authors' contributions

All authors read and approved the final manuscript. MG conceived the study, analyzed the data and drafted the paper. LF performed functional and network analyses and drafted the paper. AR, FJ, DL and JLF contributed to analyses. KMG provided samples and IG oversaw genotyping and quality control of genotyping results.

## Supplementary Material

Additional file 1 **Supplementary Figures**. This additional file is a pdf document with seven pages each corresponding to the following supplementary figures: Figure S1 (page 1): SNP Polymorphism within each population. A) Plot of heterozygosities averaged across all the 36,320 selected SNPs against the corresponding relative increase after removing non informative SNPs from the full data set B) Distribution of the number of SNPs per population for different MAF range. Figure S2 (page 2): Neighbor-Joining tree relating the 437 individuals. The tree was constructed using allele sharing distances averaged over 36,320 SNPs. Edges are colored according to the individual breed of origin. Figure S3 (page 3): Distribution of allele counts for different *F*_*IS*_, allele frequency and number of genotyped individuals. Figure S4 (page 4): Comparison of the distribution of estimated and simulated allele frequencies in the gene pool in four simulated data sets. P represents the proportion of SNP under selection simulated and Alpha the locus effect. Figure S5 (page 5): Posterior distribution of the proportion *P *of loci under selection. Distributions for the real data set and the four different simulated data sets (with known P) are represented. Figure S6 (page 6): Distribution of distances separating consecutive SNPs. Figure S7 (page 7): Decay of average pairwise *r*^2 ^with inter-marker distance for the different populations. A vertical dotted line indicates the average marker spacing of 70 kb in the study. Click here for file

Additional file 2 **Supplementary Tables**. This additional file is an excel file containing 7 sheets each corresponding to the following supplementary tables: Table S1: Differentiation between populations. Estimates of *F*_*ST *_between pairs of population computed with SMARTPCA (GENEPOP) software are below (above) the diagonal. Diagonal elements are estimates of population *F*_*IS*_. Standard deviations of the estimates are given in parenthesis. Table S2: Power to detect loci under selection as a function of the simulated locus effect. Table S3: BF and *F*_*ST *_estimates for each SNP considered in the study. Table S4: Correlation of BF and *F*_*ST *_between pair of SNPs for different inter-marker distance bins. Table S5: Description of the 7,177 genes represented by analyzed SNPs. Table S6: Description of significant gene networks. Table S7: Functional annotation of N and N3 networks. Table S8: Genome Coverage of the SNPs. Click here for file

Additional file 3 **Genotyping Data**. This file is a bzipped archive containing the genotyping data (437 individuals and 36,320 SNPs) in the Haploview format (see Methods). Click here for file
