# Supplementary material for: A whole genome Bayesian scan for adaptive genetic divergence in West African cattle
Source: BMC Genomics. 2009 Nov 21;10:550. doi: 10.1186/1471-2164-10-550 (PMC2784811; doi:10.1186/1471-2164-10-550)
Supplement: Additional file 1 — Supplementary Figures. This additional file is a pdf document with seven pages each corresponding to the following supplementary figures: Figure S1 (page 1): SNP Polymorphism within each population. A) Plot of heterozygosities averaged across all the 36,320 selected SNPs against the corresponding relative increase after removing non informative SNPs from the full data set B) Distribution of the number of SNPs per population for different MAF range. Figure S2 (page 2): Neighbor-Joining tree relating the 437 individuals. The tree was constructed using allele sharing distances averaged over 36,320 SNPs. Edges are colored according to the individual breed of origin. Figure S3 (page 3): Distribution of allele counts for different FIS, allele frequency and number of genotyped individuals. Figure S4 (page 4): Comparison of the distribution of estimated and simulated allele frequencies in the gene pool in four simulated data sets. P represents the proportion of SNP under selection simulated and Alpha the locus effect. Figure S5 (page 5): Posterior distribution of the proportion P of loci under selection. Distributions for the real data set and the four different simulated data sets (with known P) are represented. Figure S6 (page 6): Distribution of distances separating consecutive SNPs. Figure S7 (page 7): Decay of average pairwise r2 with inter-marker distance for the different populations. A vertical dotted line indicates the average marker spacing of 70 kb in the study. [file 1471-2164-10-550-S1.PDF]

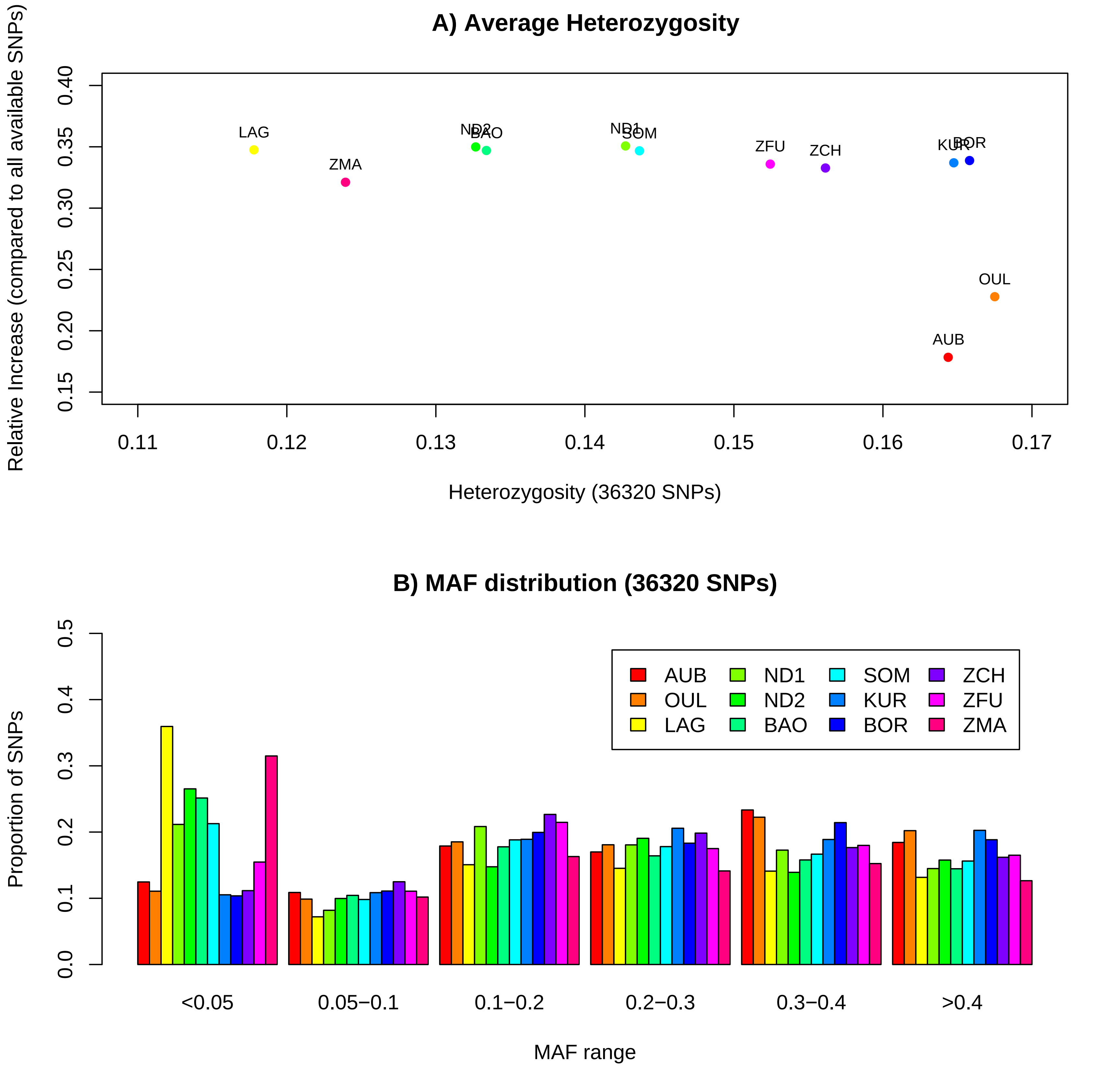
A) Average Heterozygosity
Relative Increase (compared to all available SNPs)
Heterozygosity (36320 SNPs)
0.11
0.12
0.13
0.14
0.15
0.16
0.17
0.15
0.20
0.25
0.30
0.35
0.40
LAG
ZMA
ND2
BAO
ND1
SOM
ZFU
ZCH
KUR
BOR
OUL
AUB
B) MAF distribution (36320 SNPs)
Proportion of SNPs
MAF range
0.0
0.1
0.2
0.3
0.4
0.5
<0.05
0.05−0.1
0.1−0.2
0.2−0.3
0.3−0.4
>0.4
AUB
OUL
LAG
ND1
ND2
BAO
SOM
KUR
BOR
ZCH
ZFU
ZMA

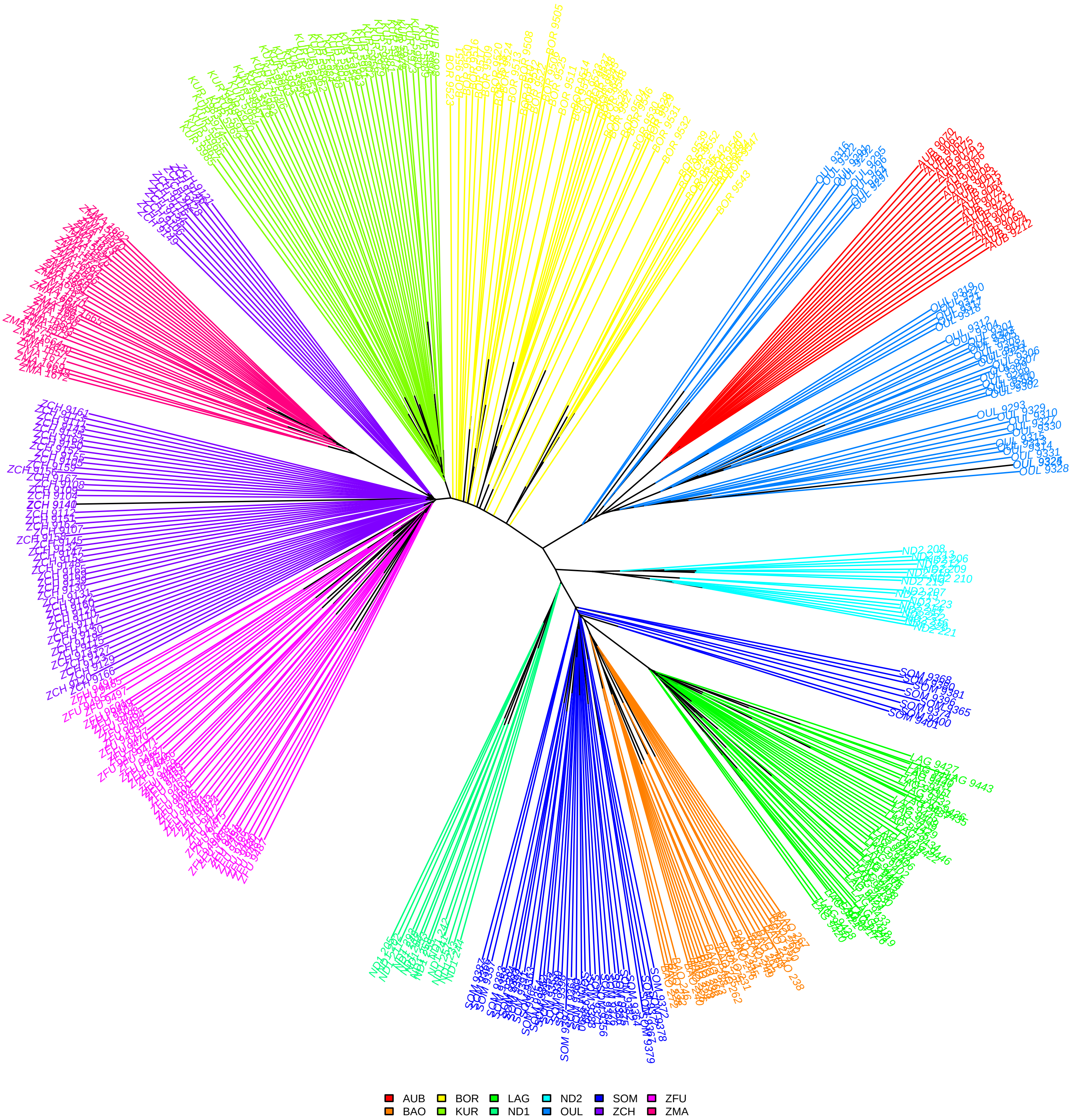
AUB
BAO
BOR
KUR
LAG
ND1
ND2
OUL
SOM
ZCH
ZFU
ZMA

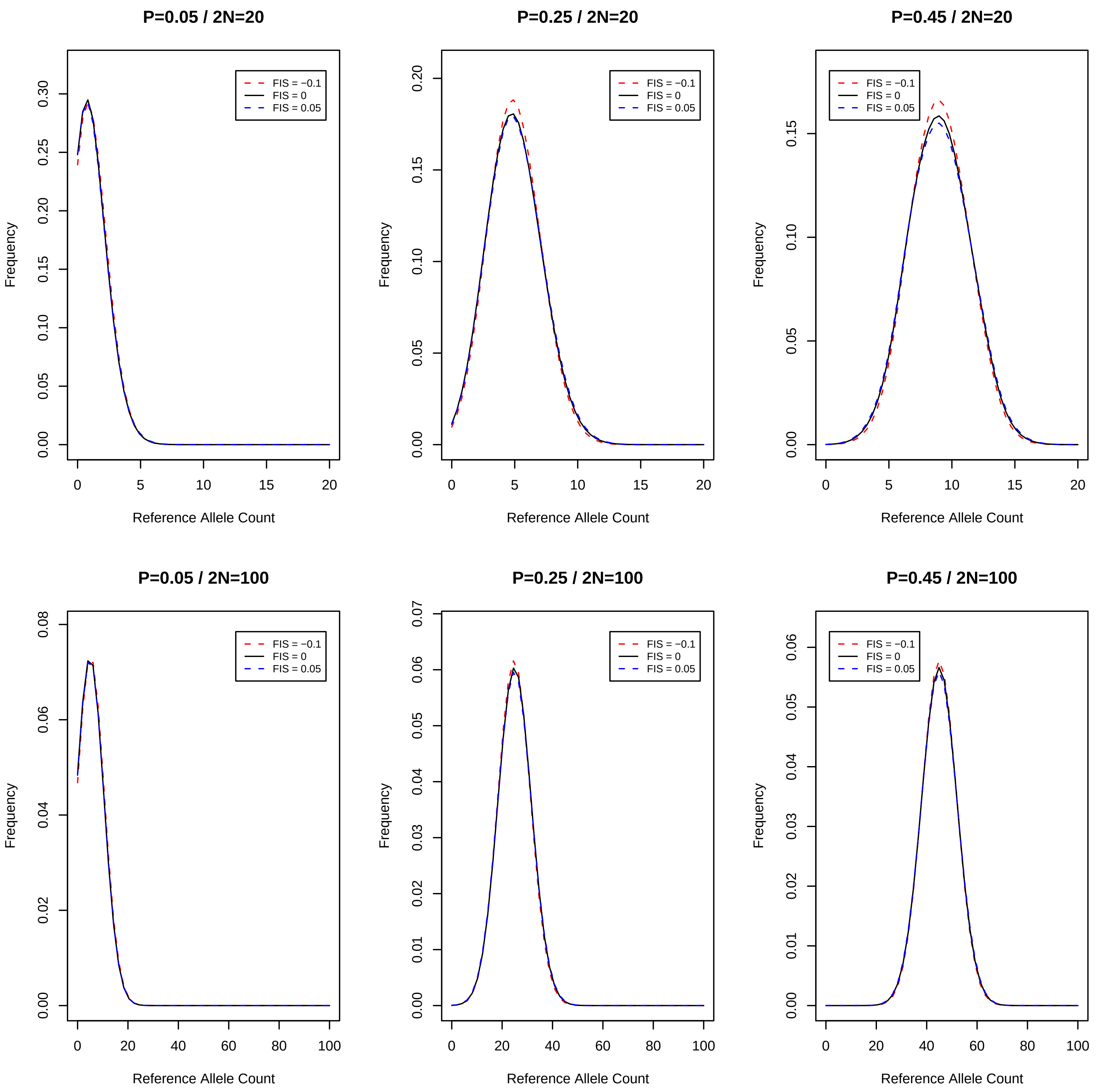
P=0.05 / 2N=20
P=0.25 / 2N=20
P=0.45 / 2N=20
P=0.05 / 2N=100
P=0.25 / 2N=100
P=0.45 / 2N=100
FIS = −0.1
FIS = 0
FIS = 0.05
Frequency
Reference Allele Count
0.00
0.05
0.10
0.15
0.20
0.25
0.30
0.01
0.02
0.03
0.04
0.06
0.07
0.08
0
5
10
15
20
40
60
80
100

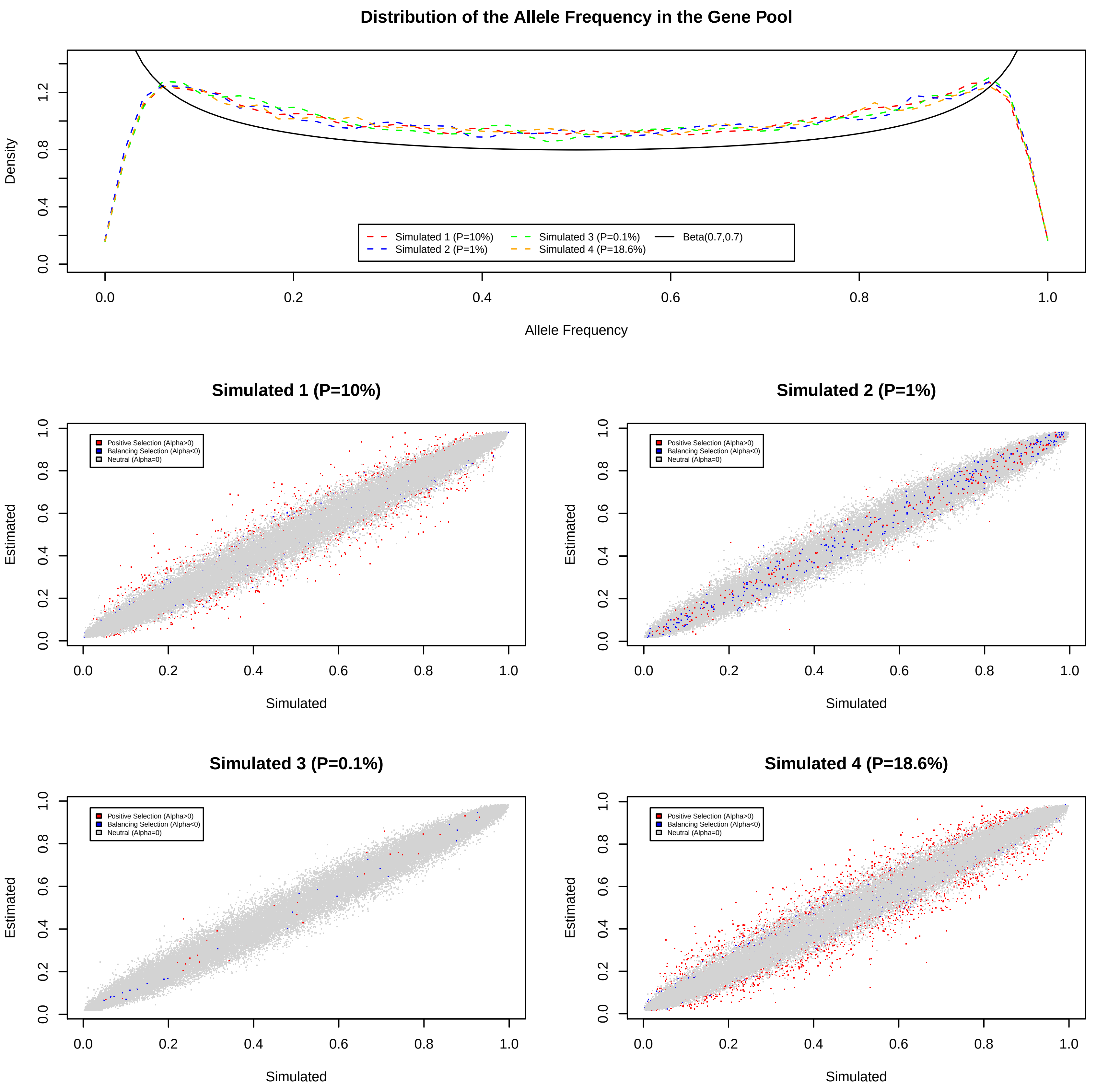
Distribution of the Allele Frequency in the Gene Pool
Density
Allele Frequency
0.0
0.4
0.8
1.2
0.0
0.2
0.4
0.6
0.8
1.0
Simulated 1 (P=10%)
Simulated 2 (P=1%)
Simulated 3 (P=0.1%)
Simulated 4 (P=18.6%)
Beta(0.7,0.7)
Simulated 1 (P=10%)
Simulated 2 (P=1%)
Simulated 3 (P=0.1%)
Simulated 4 (P=18.6%)
Positive Selection (Alpha>0)
Balancing Selection (Alpha<0)
Neutral (Alpha=0)
Estimated
Simulated
0.0
0.2
0.4
0.6
0.8
1.0

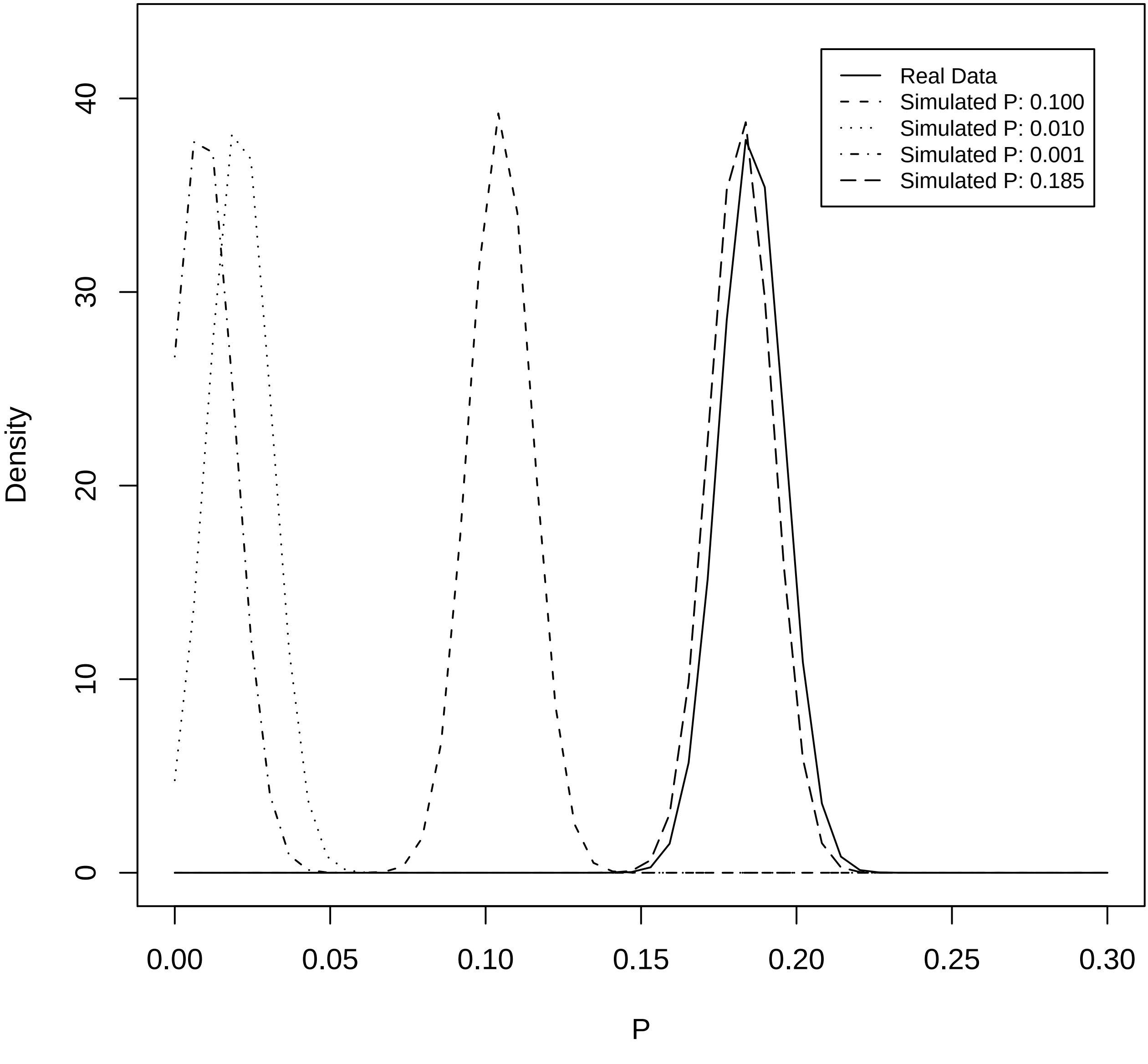

Real Data
Simulated P: 0.100
Simulated P: 0.010
Simulated P: 0.001
Simulated P: 0.185
Density
P
0
10
20
30
40
0.00
0.05
0.10
0.15
0.20
0.25
0.30

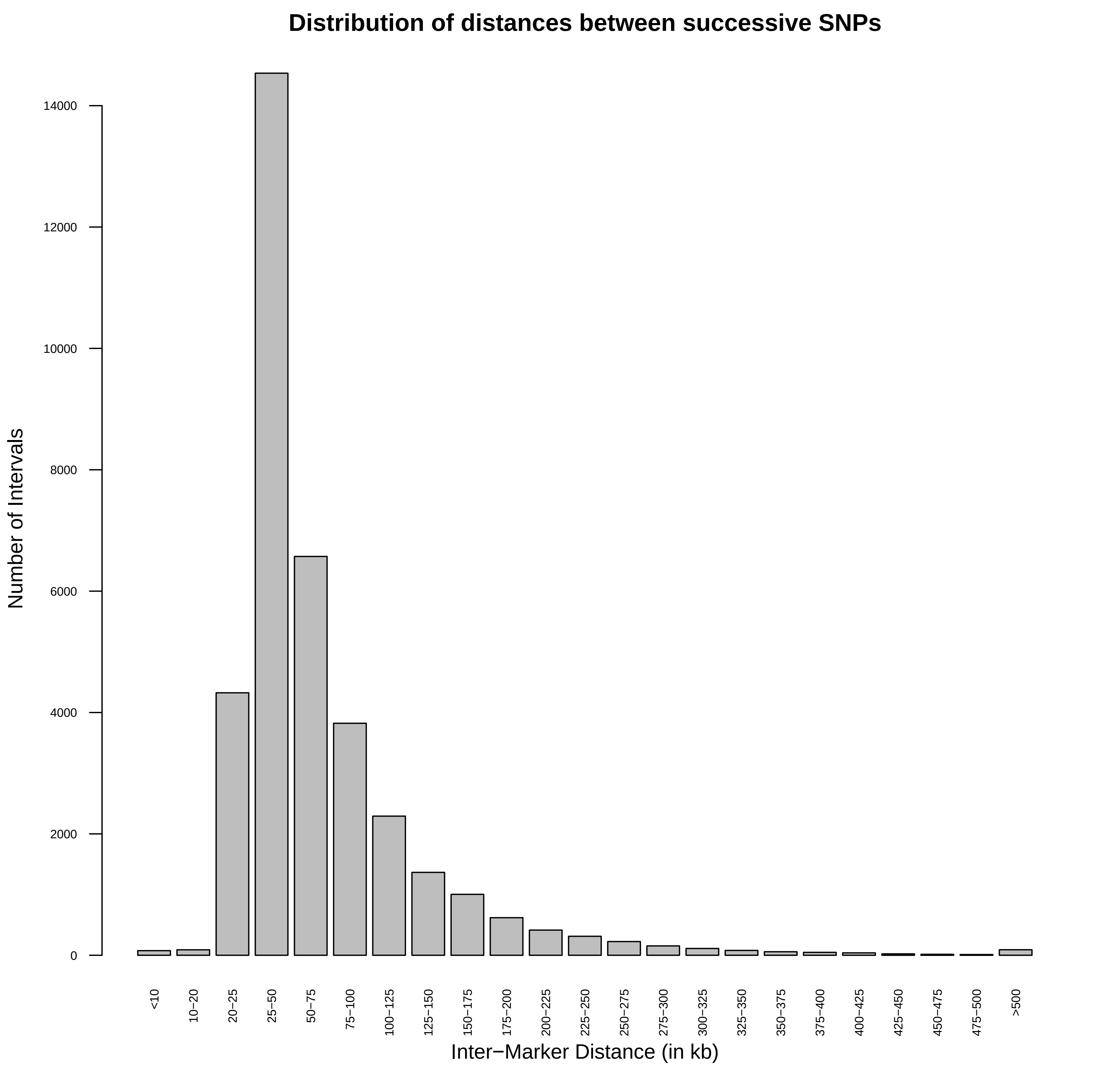
Distribution of distances between successive SNPs
Number of Intervals
0
2000
4000
6000
8000
10000
12000
14000
<10
10−20
20−25
25−50
50−75
75−100
100−125
125−150
150−175
175−200
200−225
225−250
250−275
275−300
300−325
325−350
350−375
375−400
400−425
425−450
450−475
475−500
>500
Inter−Marker Distance (in kb)

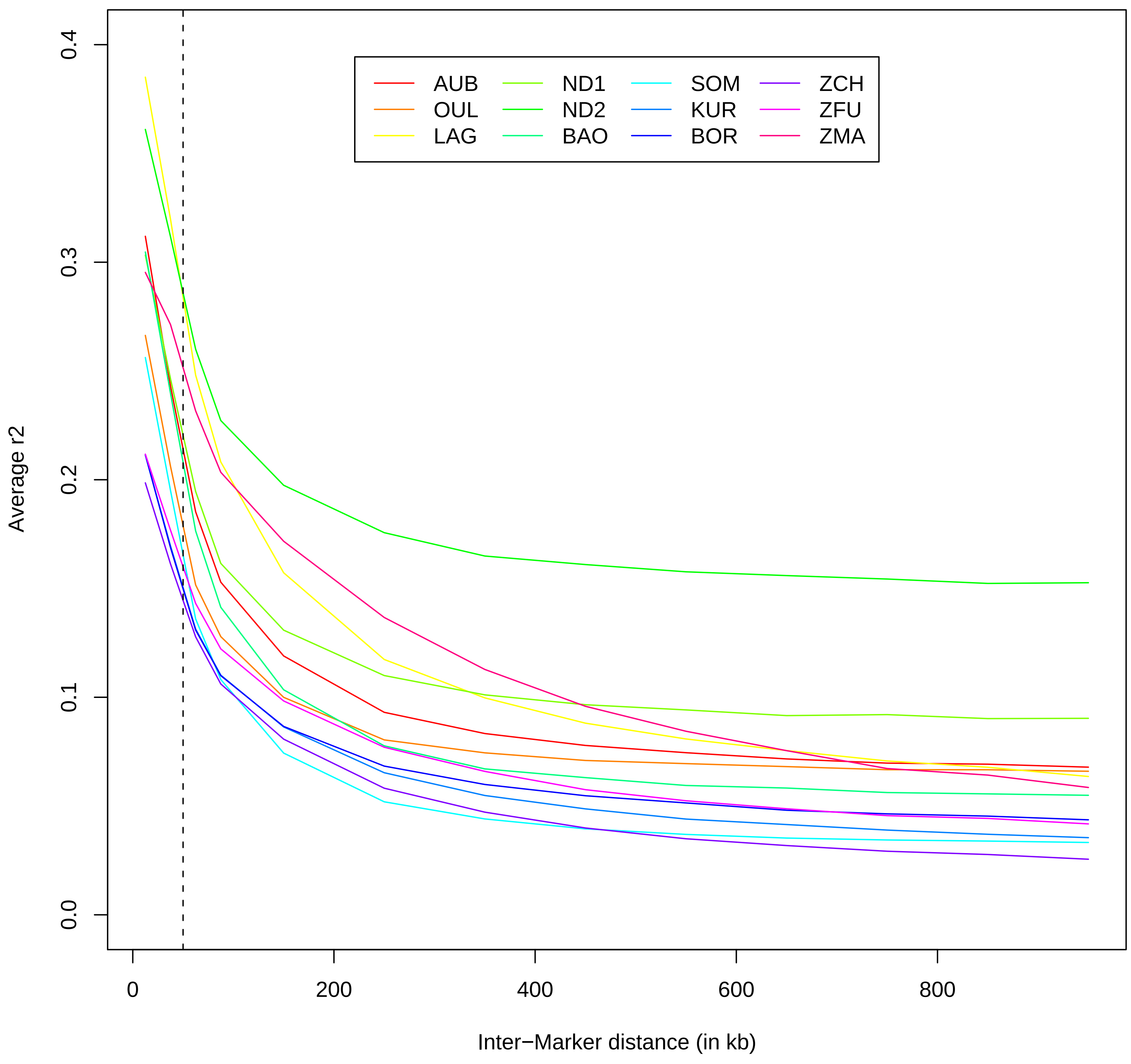

AUB
OUL
LAG
ND1
ND2
BAO
SOM
KUR
BOR
ZCH
ZFU
ZMA
Average r2
Inter−Marker distance (in kb)
0.0
0.1
0.2
0.3
0.4
0
200
400
600
800
